# In models we trust: preregistration, large samples, and replication may not suffice

**DOI:** 10.3389/fpsyg.2023.1266447

**Published:** 2023-09-21

**Authors:** Martin Spiess, Pascal Jordan

**Affiliations:** Institute of Psychology, Department of Psychology and Human Movement Science, University of Hamburg, Hamburg, Germany

**Keywords:** population, sampling design, non-response, selectivity, misspecification, biased inference, diagnostics, robust methods

## Abstract

Despite discussions about the replicability of findings in psychological research, two issues have been largely ignored: selection mechanisms and model assumptions. Both topics address the same fundamental question: Does the chosen statistical analysis tool adequately model the data generation process? In this article, we address both issues and show, in a first step, that in the face of selective samples and contrary to common practice, the validity of inferences, even when based on experimental designs, can be claimed without further justification and adaptation of standard methods only in very specific situations. We then broaden our perspective to discuss consequences of violated assumptions in linear models in the context of psychological research in general and in generalized linear mixed models as used in item response theory. These types of misspecification are oftentimes ignored in the psychological research literature. It is emphasized that the above problems cannot be overcome by strategies such as preregistration, large samples, replications, or a ban on testing null hypotheses. To avoid biased conclusions, we briefly discuss tools such as model diagnostics, statistical methods to compensate for selectivity and semi- or non-parametric estimation. At a more fundamental level, however, a twofold strategy seems indispensable: (1) iterative, cumulative theory development based on statistical methods with theoretically justified assumptions, and (2) empirical research on variables that affect (self-) selection into the observed part of the sample and the use of this information to compensate for selectivity.

## 1. Introduction

The debate around the replication crisis is not the only consequence of methodological deficiencies discussed in the psychological literature, but certainly one that has attracted a large amount of attention in recent years (e.g., Open Science Collaboration, 2012, 2015; Klein et al., 2014, 2018; Shrout and Rodgers, 2018). In fact, criticism of the methodological practice has addressed a wide range of aspects, from science policy and human bias (e.g., Sterling, 1959; Rosenthal, 1979; Sterling et al., 1995; Pratkanis, 2017) over rather general methodological approaches (e.g., Meehl, 1967, 1990; Hahn, 2011; Button et al., 2013; Fiedler, 2017) to more specific topics, like automated null hypothesis testing or underpowered studies (e.g., Rozeboom, 1960; Cohen, 1962; Sedlmeier and Gigerenzer, 1989; Gigerenzer, 2018).

The wide range of aspects criticized over a large time span suggests that most of them may be symptoms of an underlying disease rather than several isolated problems: A lack of appreciation for the close interconnection of theory and methods to analyze empirical data in psychological research. One explicit indication for an underlying nonchalant attitude is provided by Rozeboom (1960) according to whom researchers are consumers of statistical methods with the legitimate demand that the available statistical techniques meet his or her respective needs. He or she is not required to have a deeper understanding of the instruments. Rozeboom (1960) warned however, that this position makes the researcher vulnerable to misusing the tools. As discussions over time have shown, it is not enough to have a toolbox of instruments available; it must be of vital interest to researchers to know which instrument provides the relevant information under which conditions and how to interpret the results of those instruments in order to derive valid conclusions. And although more responsibility of researchers for the methods they adopt has been demanded (e.g., Hahn, 2011), this seems not to have had a strong impact on the carefulness with which statistical methods are applied and statistical results are interpreted (e.g., Gigerenzer, 2018; Fricker et al., 2019).

In this paper we consider two methodological aspects and their possible consequences in more detail that, although their possible importance has been insinuated from time to time, neither received much attention nor have been treated in more detail in the discussion of theoretical and methodological issues in psychological research: Selection of samples and handling of model assumptions (e.g., Arnett, 2008; Fernald, 2010; Henrich et al., 2010; Asendorpf et al., 2013; Falk et al., 2013; Kline, 2015; Scholtz et al., 2020).

## 2. The methodological framework

**Figure 1:**
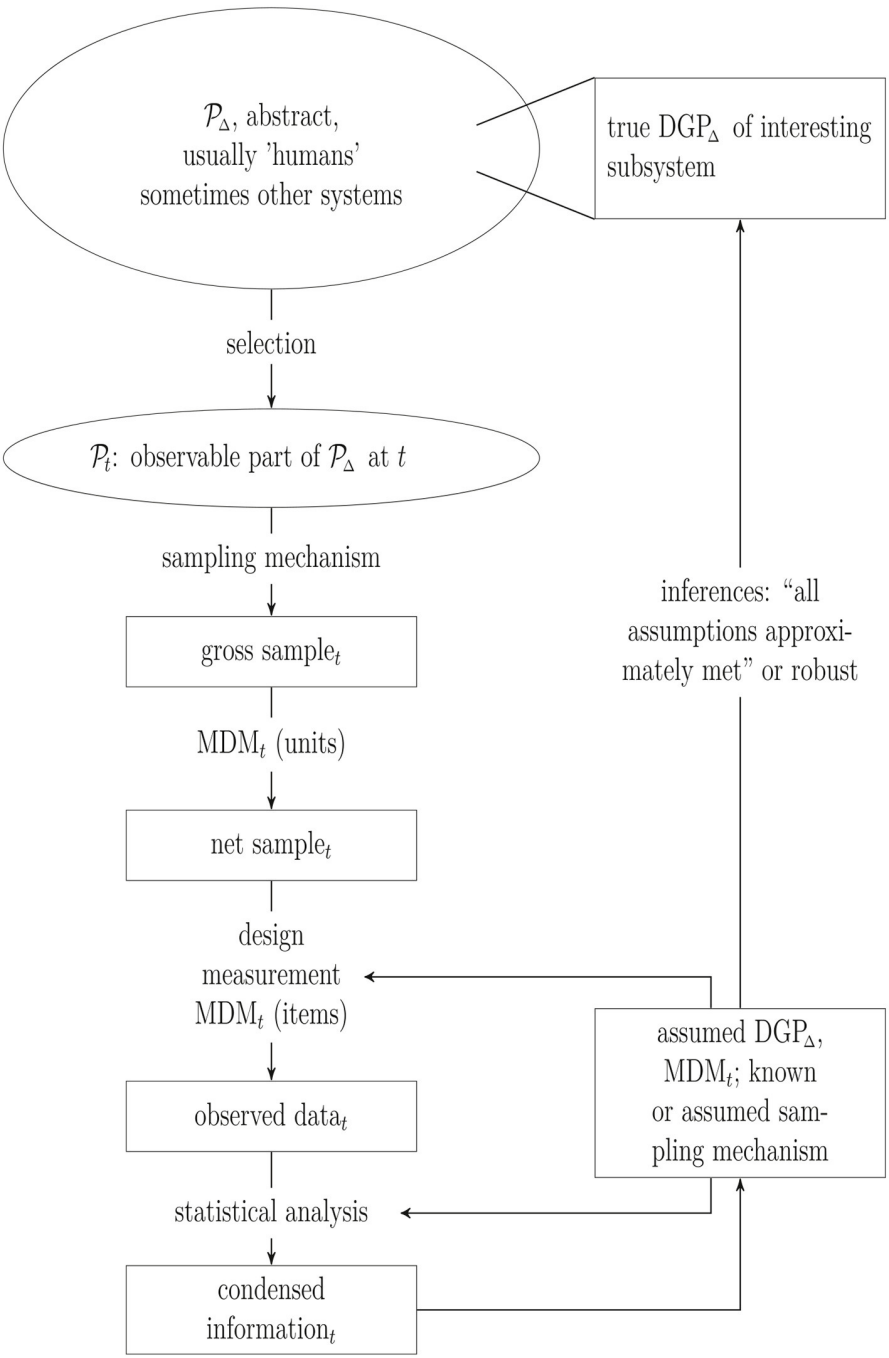
Population (${\text{𝒫}}_{△}$), sample and inference. DGP, Data generating process; MDM, missing data mechanism; *t*, time point; Δ, time interval.

The general steps from a population to the observed sample (and back) as schematically displayed in Figure 1 are not new but the graphic highlights the steps considered more closely in the subsequent sections: Selecting units from the population into the observed sample and drawing inferences from the observed sample about the assumed data generating process (DGP).

Alpha and omega of psychological research is a population of biological subsystems and, more precisely, phenomenons mostly but not exclusively related to the nervous system located in humans. The elements of the population, i.e., humans or, more generally, units, are defined by and reduced to possibly high-dimensional vector variables. For example, variables characterizing the subsystems of interest in psychological research can be indicators like socio-demographic variables, age, gender or biomarkers but also reactions evoked by some stimuli under (non-) experimental conditions. In general, however, these variables neither describe the subsystems exhaustively nor do the subsystems exist isolated. Furthermore, not the variables themselves but the process that leads to realizations at least of some of these variables, i.e., the true DGP of some variables usually given covariates or explanatory variables, is of scientific interest. However, since the units are the carriers of—among a huge number of other variables—the scientifically interesting variables, it is these units that have to be selected.

Inferences are usually intended about a DGP inevitably linked to units in a population of humans within a certain time period Δ, denoted as DGP Δ and ${\text{𝒫}}_{△}$, respectively, in Figure 1. An important criterion to evaluate the maturity of a theory is the precision with which the units and their environments can be defined. Thus, the set of humans and the time period about which inferences are intended have to be defined as clear as possible in each step of the iterative development of a theory. Are inferences intended about homo sapiens in general or about homo sapiens living in the first half of the 21st century in western, educated, industrialized, rich and democratic countries (Arnett, 2008; Henrich et al., 2010)? The answer certainly depends on the psychological subfield. For example, the intended population may be wider in general psychology as compared to social psychology. Often, however, populations are not or only vaguely defined.

In contrast to, for example, official statistics, the target population in psychological research is abstract: Inferences are made about systems linked to units that do not necessarily exist at the time the research is conducted, either because the carriers already deceased or did not yet come into existence. However, units can only be selected from an observed part of ${\text{𝒫}}_{△}$. It therefore remains part of the theory to justify that the observable subpopulation of carriers at time point *t*, ${\text{𝒫}}_{t}$, is not selective with respect to the true DGP_ Δ_ of interest.

The gross sample is the set of units selected from ${\text{𝒫}}_{t}$ by some mechanism. In official statistics this is straightforward: Select a sample of units, typically according to a predefined sampling plan, from the well-defined finite (sub)population of interest, e.g., from the residents in a given country at a defined time point. Thus, the sampling mechanism is known and is usually such that the selected or gross sample is not selective or can be corrected for its selectivity. Note that in this case ${\text{𝒫}}_{△}$ is often assumed to be approximately equal to ${\text{𝒫}}_{t}$. If the selection mechanism is unknown, then it is usually (implicitly) assumed that the selection step can be ignored in order to proceed with the analysis.

Unfortunately, there is a further selection step from the gross sample to the observed or net sample with units dropping out depending on, in most cases, an underlying unknown mechanism. For example, people belonging to ${\text{𝒫}}_{t}$ see a notice inviting them to participate in an experiment, but decide (not) to take part. This step is governed by a missing data mechanism (MDM) which at best is partly known. If enough information is available for all the units in the gross sample explaining response behavior, then it is possible to compensate for missing units. Otherwise, again, this missing information has to be replaced by the assumption that this process is not selective.

The assumed DGP Δ and, usually to a lesser extent, the assumed MDM at the item level at time *t* affect how the data are collected through the study design and measurement instruments, resulting in the observed data. This observed data set is then analyzed with statistical methods, i.e., information relevant to the research question contained in the observed data set is summarized in graphics, descriptive statistics, estimates, confidence intervals or *p*-values (“condensed information” in Figure 1).

Estimates of parameters and variances of parameters, confidence intervals or *p*-values are used to draw inferences about ${\text{𝒫}}_{t}$ and finally ${\text{𝒫}}_{△}$. These inferences will be valid if the assumed DGP Δ (approximately) correctly models how the observed data values have been generated. This requires modeling not only the true DGP Δ in ${\text{𝒫}}_{△}$ but also all (selection) processes from ${\text{𝒫}}_{△}$ to the observed data. Ignoring any of these processes is equivalent to (implicitly) assuming that they can be ignored for valid inferences in subsequent analyses and thus, statistical methods for valid inferences in simple random samples can be applied. Hence this is a modeling assumption, as is, e.g., the assumption that variables are independent from each other, that relationships are linear or that variables are normally distributed. And, of course, unjustified assumptions can easily be wrong.

Our subsequent analysis can be embedded in the different stages depicted in Figure 1. The following section will concentrate on the selection part and the missing data mechanism (*MDM*_*t*_) at the unit level, whereas Section 4 will predominantly deal with misspecifications of the (assumed) DGP. For technical details on the examples used in the text (see the Supplementary material).

## 3. Sample selection and unit response

In psychological research, samples are often selected in such a way that both, the sample selection and the unit response process are unknown and cannot be separated. An example is a convenience sample where there is no information on units that chose not to participate. Therefore, we integrate both processes into one selection mechanism. Note that the selection process can easily be generalized to cover other selection phenomena such as the file drawer problem, outlier deletion, or item non-response.

### 3.1. The general framework

Prominent examples of estimated models at the analysis stage are regression and analysis of variance models. Estimation of these models amounts to assuming a distributional model for the outcome *y* given covariates, including a 1 for the constant, collected in a vector *x*. Throughout Section 3 we presuppose that the assumed model including the required assumptions approximates the true DGP Δ sufficiently well for valid inferences.

After having selected a sample of units, it is common practice to estimate the model of DGP Δ adopting a classical model based frequentist statistical approach, using only those units whose values have been observed, with the number of observed units, *n*_obs_, and *x* fixed at their observed values. What actually should be modeled, however, is the distribution of the *y*-variables whose values have been observed given the *x*-values and the pattern of observed and not observed units from ${\text{𝒫}}_{t}$ (cf., Rubin, 1976, 1987). By conditioning on the pattern of observed and unobserved units, the selection mechanism is explicitly taken into account. Common practice is to ignore the selection mechanism, thereby implicitly assuming that it is not informative for *y* given *x*.

In regression models with independent units, it can be shown that inferences based on a model that ignores the selection mechanism will be valid if the probability of observing the actually observed units given the observed *x*-values is the same for all possible values of the observed *y* variables. See Rubin (1976) for the corresponding theory in the case of missing items. For specific models, it has also been shown that inferences about effects of covariates on the outcome ignoring the selection process are valid if the probability of the observed pattern of observed and unobserved units changes with unobserved components in *y* which are independent of *x*, but is the same for all possible values of *x* (e.g., Heckman, 1979; Terza, 1998; McCulloch et al., 2016).

On the other hand, the selection process cannot be ignored in general if for a given pattern of observed and unobserved units, the probability of observing this pattern changes with *x* and *y* even if the model correctly specifies the true DGP Δ. In this case, inferences will systematically be biased. Similar arguments hold if a non-frequentist Bayesian approach is adopted.

Given that the selection mechanism can be ignored in certain cases without biasing inferences, the question arises whether this is also true in experimental contexts, which are considered the silver bullet for unbiased causal inference.

### 3.2. Selectivity in experimental designs

One way to model the selection process is through a threshold model,


(1) $$v_{i}^{*}=\;z_{i}^{T}\gamma +w_{i}\;,\;w_{i}\sim N(0,\sigma_{w}^{2})\text{ and }v_{i}=\{\begin{array}{ll} 1 & \text{if }v_{i}^{*}\leq c\\ 0 & \text{otherwise,} \end{array}$$

wherein *z*_*i*_ is a vector of covariates including a 1 for the constant and possibly (elements of) *x*_*i*_ or $x_{i^{\prime }}$ (*i*≠*i*′), $z_{i}^{T}\gamma =\gamma_{0}+z_{i,1}\gamma_{1}+z_{i,2}\gamma_{2}+\cdots \;$ , and $v_{i}^{*}$ is an unobserved tendency to observe unit *i*, such that *y*_*i*_ and *x*_*i*_ are only completely observed if $v_{i}^{*}\leq c$, *i* = 1, …, *N*, in which case the response indicator *v*_*i*_ takes on the value one. Otherwise, if the unit is not observed, *v*_*i*_ = 0. Large values of γ model strong impacts of the covariates in *z*_*i*_ on the probability of (not) observing unit *i* in the sample. The unknown threshold *c* regulates the fraction of observed units: High values of *c* lead to high percentages of observed units and low values to small fractions. For simplicity, we assume $w_{i}\sim N\left(0,\sigma_{w}^{2}\right)$, i.e., that the error term *w*_*i*_ is normally distributed with mean zero and variance $\sigma_{w}^{2}$, not depending on *z*_*i*_ or $z_{i^{\prime }}$.

**Figure 2:**
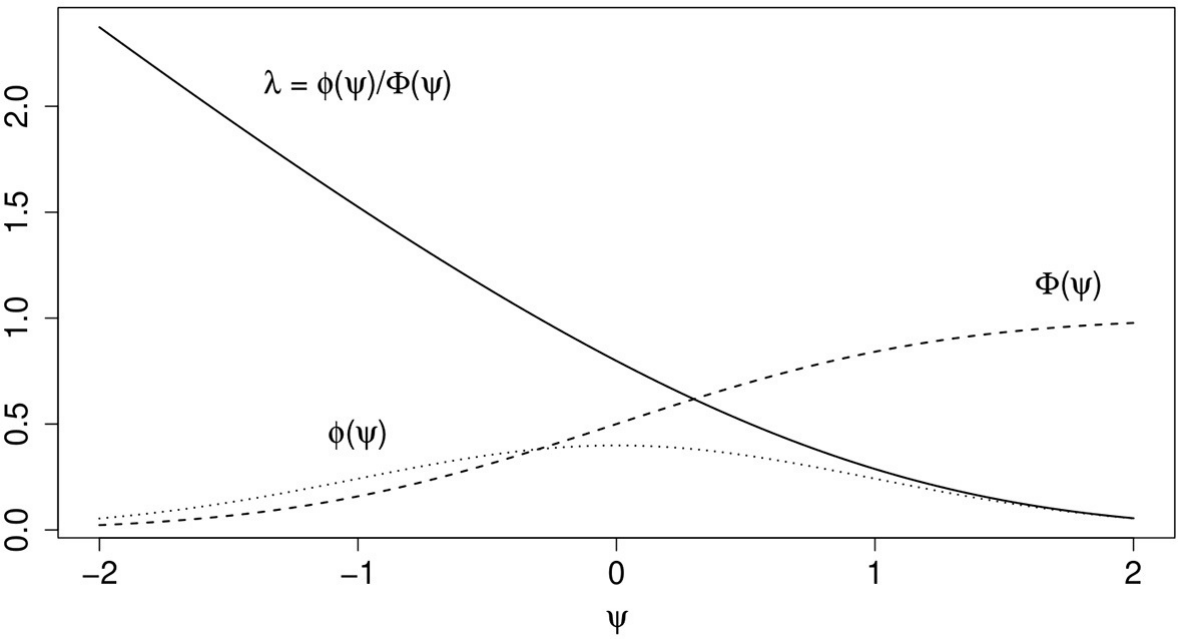
Illustration of effects of $\psi =\left(c-z^{T}\gamma \right)/\sigma_{w}$, wherein *c* = 0, *z*, and γ are both scalars and γ = σ_*w*_ = 1, on ϕ(ψ), Φ(ψ), and λ = ϕ(ψ)/Φ(ψ).

Based on these assumptions, let


$$\begin{array}{l} \psi_{i}=\frac{c-z_{i}^{T}\gamma }{\sigma_{w}}\;\text{and}\;\lambda_{i}=\frac{\phi \left(\psi_{i}\right)}{\Phi \left(\psi_{i}\right)}, \end{array}$$

wherein ϕ(·) and Φ(·) are the density and standard normal distribution function, respectively. The term ψ_*i*_ can be interpreted as the expected tendency to be selected into the sample and to respond, Φ(ψ_*i*_) models the probability that unit *i* is observed and λ_*i*_ is a term that corrects for the selection mechanism in the model of scientific interest (cf.Heckman, 1979; Amemiya, 1985). Figure 2 illustrates the effect of ψ on ϕ(·), Φ(·), and λ.

To illustrate the consequences of ignoring the selection mechanism for inference in experimental settings, we consider three examples in two scenarios that amount to a comparison of means in two groups.

#### 3.2.1. Scenario 1: one measurement per unit

Assume that the correctly specified model for the true DGP Δ is


(2) $$\begin{array}{l} y_{i}=x_{i}^{T}\beta +\epsilon_{i},\;\epsilon_{i}\sim N\left(0,\sigma_{\epsilon }^{2}\right),\;i=1,\ldots ,N, \end{array}$$

wherein β is the parameter of interest, the errors ϵ_*i*_ are independent across all units and all assumptions for valid inferences are met in ${\text{𝒫}}_{t}$, and let ϵ_*i*_ and *w*_*i*_ follow a bivariate normal distribution with correlation 0 ≤ ρ_ϵ, *w*_ < 1. Hence, we may write $\epsilon_{i}=\rho_{\epsilon ,w}\sigma_{\epsilon }\sigma_{w}^{-1}w_{i}+\zeta_{i}$, wherein ζ_*i*_ is normally distributed with mean zero and variance $\sigma_{\epsilon }^{2}\left(1-\rho_{\epsilon ,w}^{2}\right)$ (e.g., Mardia et al., 1979).

Taking the selection process into account, the model of scientific interest that would have to be estimated based on the observed sample is a model for *y*_*i*_ conditional on *x*_*i*_ as a function of *w*_*i*_ which, in the observed sample, i.e., for units with $w_{i}\leq c-z_{i}^{T}\gamma$, is truncated above at $c-z_{i}^{T}\gamma$ and thus follows a truncated normal distribution. Let *i* = 1, …, *n*_obs_ index the units in this subsample. Following Heckman (1979), for *i* = 1, …, *n*_obs_, the model to be estimated is


(3) $$\begin{array}{l} y_{i}=x_{i}^{T}\beta -\rho_{\epsilon ,w}\sigma_{\epsilon }\lambda_{i}+{\tilde{\epsilon }}_{i}, \end{array}$$

wherein $E\left({\tilde{\epsilon }}_{i}|x_{i},z_{i},v_{i}=1\right)=0$ and the term ρ_ϵ, *w*_σ_ϵ_λ_*i*_ corrects for a possible bias due to the selection process.

Let $x_{i}={\left(1x_{i}\right)}^{T}$ where *x*_*i*_ is a binary variable, resulting in a comparison of the means of two independent groups defined by *x*_*i*_ = 0 and *x*_*i*_ = 1, respectively. Ignoring the selection mechanism, which is equivalent to ignoring the term ρ_ϵ, *w*_σ_ϵ_λ_*i*_, leads to the estimator of the difference in the means of the two groups,


(4) $$\begin{array}{l} \;{\hat{\beta }}_{\mu_{1}-\mu_{0}}={\bar{y}}_{1}-{\bar{y}}_{0}\text{ and}\\ \text{𝔼}({\hat{\beta }}_{\mu_{1}-\mu_{0}}|x_{\text{obs}},v_{\text{obs}})\;=(\mu_{1}-\mu_{0})-\rho_{\epsilon ,w}\sigma_{\epsilon }({\bar{\lambda }}_{1}-{\bar{\lambda }}_{0})\;, \end{array}$$

wherein ȳ_0_ and ȳ_1_ are the sample means and μ_0_ and μ_1_ are the true population means of *y*-values for which *x*_*i*_ = 0 and *x*_*i*_ = 1, respectively, ${\bar{\lambda }}_{0}$ is the sample mean of λ_*i*_ values if *x*_*i*_ = 0 and ${\bar{\lambda }}_{1}$ is the sample mean of λ_*i*_ values if *x*_*i*_ = 1. Thus, the bias of the estimator for the difference between the two groups, μ_1_−μ_0_, is $-\rho_{\epsilon ,w}\sigma_{\epsilon }\left({\bar{\lambda }}_{1}-{\bar{\lambda }}_{0}\right)$.

The estimator of μ_1_−μ_0_ will be biased if ρ_ϵ, *w*_≠0, i.e., if there is at least one variable, independent from *x*_*i*_ and *z*_*i*_, that has an effect on the selection process and is linearly related to the outcome in the model of scientific interest, and if the difference ${\bar{\lambda }}_{1}-{\bar{\lambda }}_{0}$ is not zero. This latter difference is not zero if the tendencies to be observed in the sample differ systematically between the subsamples defined by *x*_*i*_ = 0 and *x*_*i*_ = 1, respectively. Any bias will be amplified by a decreasing fit of the model of scientific interest. Note that even if the two population means μ_0_ and μ_1_ are equal, the estimator of their difference may systematically be different from zero.

If the assignment of each unit to one and only one condition is random and independent from *x*_*i*_, ${\bar{\lambda }}_{1}-{\bar{\lambda }}_{0}$ will usually (approximately) be zero and thus the estimator of the mean difference between the groups will (approximately) be unbiased. In this case, the selection mechanism can be ignored even if selection into the observed part of the sample depends on variables that have an effect on the outcome.

However, if the selection mechanism depends on *x*_*i*_, then ${\bar{\lambda }}_{1}-{\bar{\lambda }}_{0}$ will not (approximately) be zero because the bounds $c-z_{i}^{T}\gamma$ will systematically be different in the two groups. For example, suppose that the two levels of *x*_*i*_ represent two clinical groups that differ in their willingness to participate in a study, e.g., because of a decreased level of physical activity in one of the two groups, that affects the outcome only through *x*_*i*_. If in addition there are variables, like general openness, independent from *x*_*i*_ and *z*_*i*_ that affect both, the outcome of interest and the tendency to be observed in the sample, so that ρ_ϵ, *w*_≠0, then the estimator for the difference in the means in ${\text{𝒫}}_{t}$ will be biased and inferences will be invalid. Ignoring the ρ_ϵ, *w*_σ_ϵ_λ_*i*_ part is equivalent to estimating a misspecified model, although the model would be correctly specified in ${\text{𝒫}}_{t}$.

**Figure 3:**
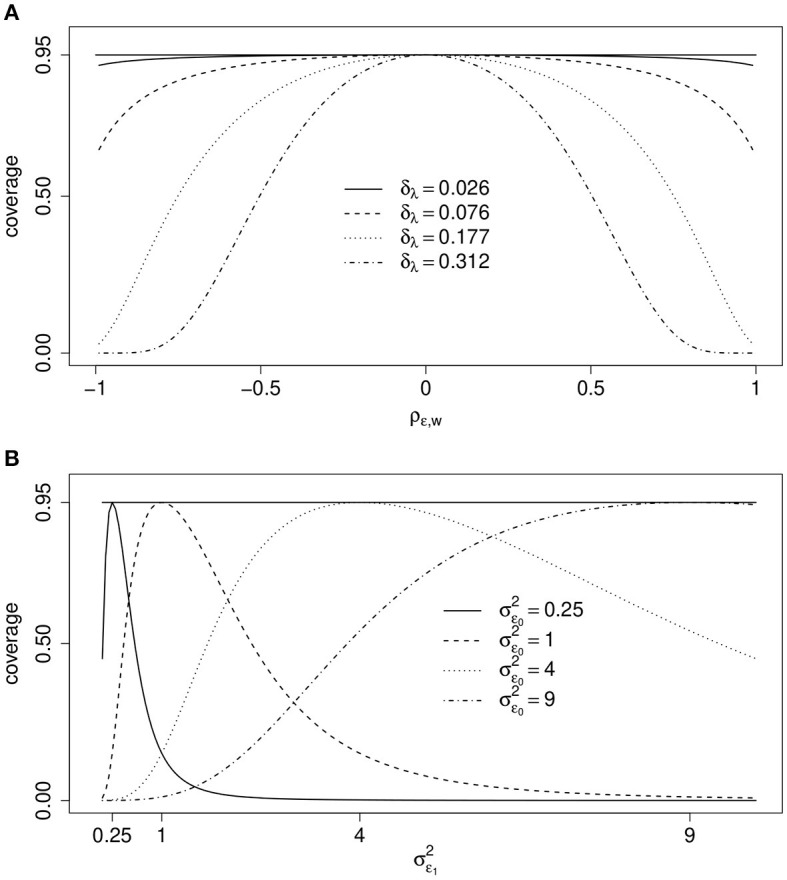
One measurement per unit. Coverage rate (coverage) of true value μ_1_−μ_0_ = 0 by 0.95-confidence intervals as a function of, **(A)** ρ_ϵ, *w*_ and different values of $\delta_{\lambda }={\bar{\lambda }}_{1}-{\bar{\lambda }}_{0}$ if ρ_*z, x*_ = 0.5 and σ_ϵ_ = 1, and, **(B)** $\sigma_{\epsilon_{1}}^{2}$ and different values of $\sigma_{\epsilon_{0}}^{2}$ if ρ_ϵ, *w*_ = 0.4, γ = 0 and δ_λ_ = 0 (see text for details). In both scenarios $\sigma_{w}^{2}=1$.

Figure 3 illustrates the effect of ρ_ϵ, *w*_ on the coverage rate of the true values μ_1_−μ_0_ = 0 based on 0.95-confidence intervals if $\sigma_{\epsilon }^{2}=1$, *x*_*i*_ and a scalar binary *z*_*i*_ are correlated with ρ_*z, x*_ = 0.5, and for different values of $\delta_{\lambda }={\bar{\lambda }}_{1}-{\bar{\lambda }}_{0}$. If the correlation ρ_ϵ, *w*_ or the difference of the means of ${\bar{\lambda }}_{1}$ and ${\bar{\lambda }}_{0}$ is close to zero, then the actual coverage rate of the true difference of the two means is close to the nominal level 0.95. The actual coverage rate decreases, however, with increasing values of δ_λ_ or ρ_ϵ, *w*_ if both are not zero. The actual coverage rate of the 0.95-confidence interval can drop even below 0.5, leading to rejection rates of the true null hypothesis that are far too high. Thus, a non-existing effect may be “found” far too often.

For the second example, we introduce a minor change: Assume possibly different error variances under the two conditions in DGP Δ, i.e., $\sigma_{\epsilon_{0}}^{2}$ if *x*_*i*_ = 0 and $\sigma_{\epsilon_{1}}^{2}$ if *x*_*i*_ = 1. For simplicity we assume ρ_ϵ_0_, *w*_ = ρ_ϵ_1_, *w*_. Then, the expected value of ${\hat{\beta }}_{\mu_{1}-\mu_{0}}$ ignoring the selection mechanism is


(5) $$\begin{array}{l} \text{𝔼}({\hat{\beta }}_{\mu_{1}-\mu_{0}}|x_{\text{obs}},v_{\text{obs}})=(\mu_{1}-\mu_{0})-\rho_{\epsilon ,w}(\sigma_{\epsilon_{1}}{\bar{\lambda }}_{1}-\sigma_{\epsilon_{0}}{\bar{\lambda }}_{0}). \end{array}$$

Thus, the estimator for the difference μ_1_−μ_0_ will generally be biased if there is any variable independent of *z*_*i*_ and *x*_*i*_ that has an effect on selection and *y*_*i*_, and if $\sigma_{\epsilon_{0}}^{2}\neq \sigma_{\epsilon_{1}}^{2}$ even if assignment to the two conditions is random and does not depend on *x*_*i*_.

Figure 3 illustrates the coverage rates of true value μ_1_−μ_0_ = 0 by 0.95-confidence intervals under this more general scenario. Now ρ_ϵ, *w*_ = 0.4, δ_λ_ = 0 and selection does not depend on *z*_*i*_ = *z*_*i*_ because γ = 0. What varies are the error variances $\sigma_{\epsilon_{0}}^{2}$ and $\sigma_{\epsilon_{1}}^{2}$. The actual coverage rates are equal to the nominal 0.95-level if both error variances are equal but differ greatly for large differences between the two. Again, effects may be “found” much too often even if μ_1_ = μ_0_.

#### 3.2.2. Scenario 2: two measurements per unit

Consider a repeated measurement design, where each unit is observed under each of two conditions, *x*_*i*_ = 0 and *x*_*i*_ = 1, but the selection mechanism is given by Equation (1). We further assume that there are no systematic position effects. Using the same notation and estimator for the difference μ_1_−μ_0_ as in the last section, its expected value ignoring the selection process is


$$\begin{array}{l} \text{𝔼}({\hat{\beta }}_{\mu_{1}-\mu_{0}}|x_{\text{obs}},v_{\text{obs}})=(\mu_{1}-\mu_{0})-\bar{\lambda }(\rho_{\epsilon_{1},w}\sigma_{\epsilon_{1}}-\rho_{\epsilon_{0},w}\sigma_{\epsilon_{0}}), \end{array}$$

wherein ρ_ϵ_0_, *w*_ and ρ_ϵ_1_, *w*_ are the correlations of the errors in the model of scientific interest with *w*_*i*_ in Equation (1), respectively, and σ_ϵ_0__ and σ_ϵ_1__ are the corresponding variances. Because $\bar{\lambda }$ is not zero if there are unobserved units, the estimator of μ_1_−μ_0_ is biased if ρ_ϵ_0_, *w*_σ_ϵ_0__≠ρ_ϵ_1_, *w*_σ_ϵ_1__. Hence, if there is any variable independent from *x* and *z* which is not included into the model of scientific interest but has different effects on *y*_0_ and *y*_1_ and is relevant in the selection and response mechanism, then the estimator of the difference μ_1_−μ_0_ will be biased and corresponding inferences will not be valid. The amount of bias will be amplified by decreasing values of *c* or, for positive γ, by increasing values of *z* and thus by larger values of $\bar{\lambda }$.

**Figure 4:**
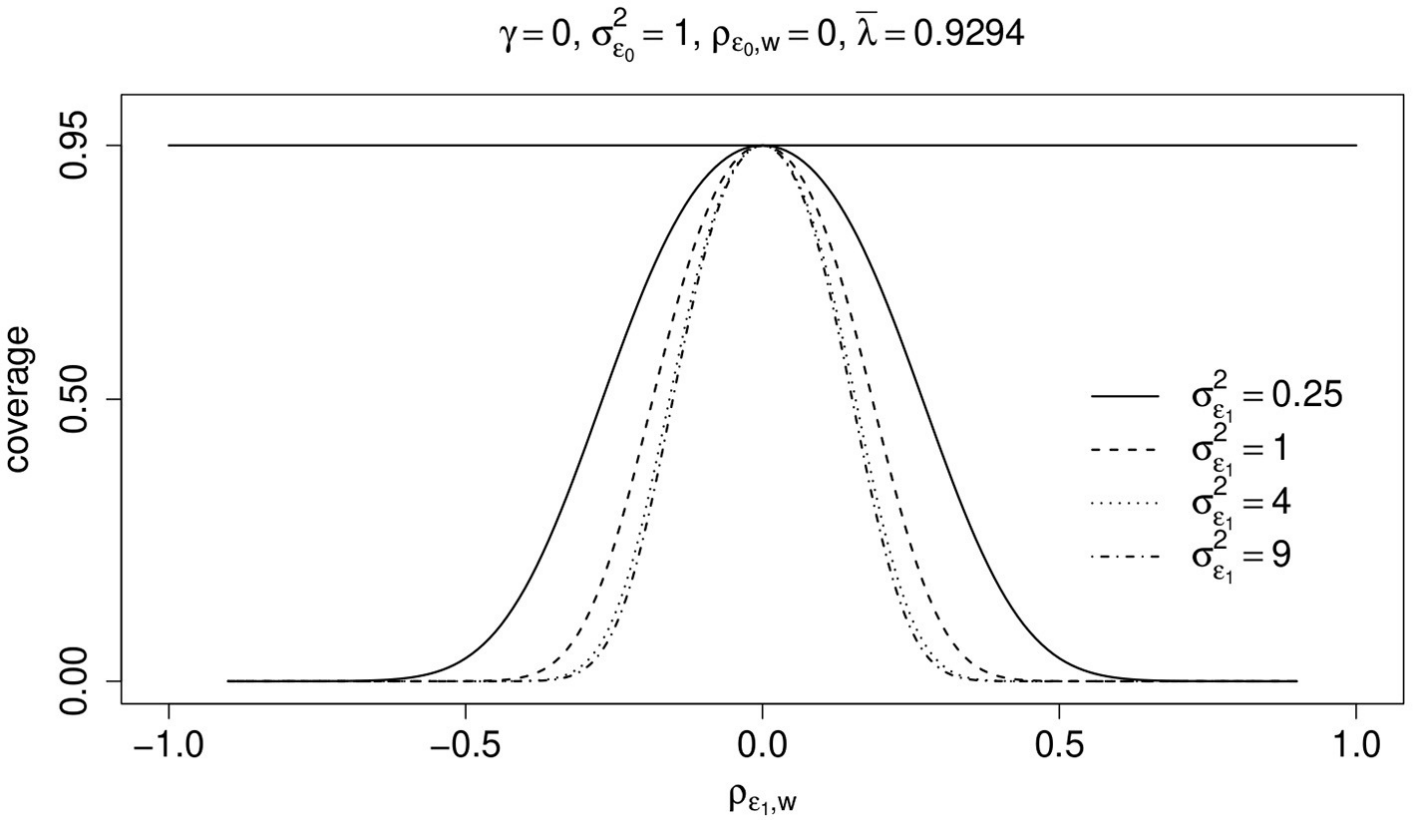
Two measurements per unit. Coverage rate (coverage) of true value μ_1_−μ_0_ = 0 by 0.95-confidence intervals as a function of ρ_ϵ_1_, *w*_ and different values of $\sigma_{\epsilon_{1}}^{2}$. Covariance of ϵ_0_ and ϵ_1_ is $\sigma_{\epsilon_{0}\epsilon_{1}}^{2}=0.2$ (see text for details).

Figure 4 shows, for different values of $\sigma_{\epsilon_{1}}^{2}$, the effect of ρ_ϵ_1_, *w*_ on the actual coverage rate of 0.95-confidence intervals. Again, there is only one *z*_*i*_-variable the corresponding parameter of which is zero, i.e., γ = 0. For simplicity, ρ_ϵ_0_, *w*_ is zero and σ_ϵ_0__ = 1. The mean over all λ_*i*_-values in the observed sample is $\bar{\lambda }=0.9294$ and the covariance of ϵ_0_ and ϵ_1_, $\sigma_{\epsilon_{0}\epsilon_{1}}^{2}$, is 0.2. Thus, the bias is not zero and increases with increasing (absolute) values of ρ_ϵ_1_, *w*_ and $\sigma_{\epsilon_{1}}^{2}$. Consequently, the actual coverage rate may dramatically decline with increasing (absolute) values of ρ_ϵ_1_, *w*_ and $\sigma_{\epsilon_{1}}^{2}$. If ρ_ϵ_1_, *w*_ is zero, then the actual coverage rates are equal to the nominal 95%-coverage rate.

As an example, consider a simple reaction time experiment with two conditions, and suppose students at a university are invited to participate. If age is an indicator of the developmental stage of a subsystem related to reaction time, and if the disregarded age affects the outcome variable reaction time differently under the two conditions via the corresponding subsystem (e.g., Dykiert et al., 2012), then ignoring the selection mechanism will lead to biased inferences. In this simplified example disregarded age would be part of *w*, which would be correlated with ϵ_0_ and ϵ_1_.

## 4. Violations of model assumptions

In this section we assume that the selection of units can be ignored. Instead, we discuss the consequences of model misspecifications in more general models commonly used in applications, but without further detailed examples.

### 4.1. Ordinary linear regression models

Suppose that different studies addressing the same research topic possibly differ in the (implicit) subpopulation they are referring to and that our (perhaps meta analytical) aim might be to infer effects in an appropriately defined mixture population. To sketch the possible inconsistency issues that might result, we assume the following: The aim is to infer the effect of some predictor variable *x* on some outcome *y* in ${\text{𝒫}}_{t}$, which can, for the sake of simplicity, be subdivided into two subpopulations, *k* = 1, 2. Assuming that the modeling assumptions hold in each subpopulation, we will analyze under what conditions these modeling assumptions hold in the mixture.

We thus take a sample (*y*_*i*_, *x*_*i*_), *i* = 1, …*n*, from ${\text{𝒫}}_{t}$ and ask whether the standard assumptions (see the Supplementary material) along with the normality assumption also hold within the mixture. To this end, abbreviate by *z*_*i*_ now the random variable which denotes the subpopulation to which the *i*-th unit belongs. According to our assumptions, we have


(6) $$\begin{array}{l} 𝔼\left(y|x,z\right)=\beta_{0}\left(z\right)+x\beta_{1}\left(z\right), \end{array}$$

wherein the intercept β_0_(*z*) and slope β_1_(*z*) may depend on the subpopulation *z*. Equation (6) entails a linear regression of *y* on *x* within each subpopulation whereby the regression lines might differ across the subpopulations. If they differ, then there is an interaction between *z* and *x* with respect to the outcome.

According to the law of iterated expectation, it follows that our key term of interest—the conditional expectation in the mixture population—is given by


(7) $$\begin{array}{l} \text{𝔼}(y|x)\;=\;{\text{𝔼}}_{z|x}(\text{𝔼}(y|x,z)|x)={\text{𝔼}}_{z|x}(\beta_{0}(z)+x\beta_{1}(z)|x)\\ \;={\text{𝔼}}_{z|x}(\beta_{0}(z)|x)+x{\text{𝔼}}_{z|x}(\beta_{1}(z)|x)=g_{0}(x)+xg_{1}(x), \end{array}$$

wherein we use *E*_*z*|*x*_ as a shorthand notation to indicate the conditional distribution of *z* given *x* with respect to which the expectation has to be taken.

We may now distinguish between three cases: Firstly, independence of *x* and *z*. Here, the conditional expectations with respect to *E*_*z*|*x*_ resolve to unconditional expectations and we arrive at


$$\begin{array}{l} {\text{𝔼}}_{z|x}(\beta_{0}(z)|x)+x{\text{𝔼}}_{z|x}(\beta_{1}(z)|x)=\beta_{0}+x\beta_{1}, \end{array}$$

wherein both $\bar{\beta }$ parameters are weighted averages of the subpopulation specific intercept and slope terms. Therefore, although the regression parameters differ from those in the subpopulations, the presumed functional form in the modeling of *E*(*y*|*x*) remains identical to the form which was assumed within each subpopulation.

Secondly, lack of interaction. In this case, the intercept and slope terms do not depend on *z* and Equation (7) reduces to:


$$\begin{array}{l} {𝔼}_{z|x}\left(\beta_{0}\left(z\right)|x\right)+x{𝔼}_{z|x}\left(\beta_{1}\left(z\right)|x\right)=\beta_{0}+x\beta_{1}. \end{array}$$

Again, the functional form is preserved and in this case also the parameters.

Thirdly, interaction or dependency. Then the conditional expectation is a function of *x* and we may conclude that the conditional expectation is furthermore likely to include nonlinear terms despite the fact that within each subpopulation we have linearity. Or stated differently: Suppose we are given two publications on the impact of the predictor *x* on the outcome *y*. Assuming the validity of the assumptions in each study, we infer the impact of the predictor via the regression coefficient β_1_. However, if a third researcher conducts a study in the mixture population, which would be a natural setup to draw meta analytical conclusions, then to ensure the validity of a linear regression model, the researcher would have to deviate from the model used in the publications. In addition, the report of the impact of the predictor would have to focus on different coefficients.

The described dependencies of the modeling assumptions on the population as well as on the sampling scheme were highlighted in terms of the ordinary linear regression model which just served as a mathematical convenient example to demonstrate these effects. The described phenomena occur in more complicated modeling classes as well, as illustrated in the following section.

### 4.2. Generalized linear mixed model (GLMM)

The class of GLMMs has many applications in psychology, most notably in Item Response Theory (IRT). As literally every construct of interest in psychology requires an adequate measurement device, it is hardly an overstatement to say that IRT models, alongside with their older classical test theory counterparts,^1^ are omnipresent in applied research. For the sake of clarity, we will therefore limit the statement of the model to the most relevant case of IRT and refer for general formulation of the GLMM to Jiang (2007). We will also exclude any covariates in order to focus on the random effects part of the model that goes beyond the ordinary regression setup.

Let *y*_*i, j*_ denote the response of the *i*-th test taker to item *j* (*j* = 1, …, *J*) of a test that is supposed to measure a single construct—say numerical IQ, denoted by θ_*i*_. The response *y*_*i, j*_ is binary, encoding as to whether the item was solved correctly or not. The postulate of a single underlying construct when combined with the local independence assumption^2^ provides us with a formula for the probability of any particular response pattern on the test, for example:


(8) $$\begin{array}{l} P\left(y_{i,1}=1,y_{i,2}=0,\ldots y_{i,k}=0|\theta \right)=f_{1}\left(\theta \right)\left(1-f_{2}\left(\theta \right)\right)\cdots \left(1-f_{k}\left(\theta \right)\right), \end{array}$$

wherein *f*_*j*_(·) denotes the item response function (IRF) of the *j*-th item. The latter is defined as the conditional probability that a test taker with latent ability θ_*i*_ = θ solves the *j*-th item, i.e., *f*_*j*_(θ) = *P*(*y*_*i, j*_ = 1|θ).

There are two key parts, wherein restrictive modeling assumptions emerge: Firstly, the IRF must be specified, leading to particular assumptions such as imposing logistic shape on *f*_*i*_. Thus, *f*_*i*_ has a given shape but may depend on a few remaining parameters—such as item difficulty and discrimination parameter(s)—which are suppressed in our notation. Secondly, apart from the special case of a Rasch model (Andersen, 1970), one needs to specify a distribution for the latent variable. This is necessary because the conditional probabilities being functions of unknown latent abilities in Equation (8) are not amenable.

However, by using the law of total probability in conjunction with the specification of a distribution function for θ, Equation (8) resolves to an empirical testable statement referring to observable quantities with no hidden quantities involved,


(9) $$\begin{array}{l} P\left(y_{i,1}=1,y_{i,2}=0,\ldots y_{i,k}=0\right)\\ =\underset{\theta }{\sum }f_{1}\left(\theta \right)\left(1-f_{2}\left(\theta \right)\right)\cdots \left(1-f_{k}\left(\theta \right)\right)P\left(\theta \right). \end{array}$$

In the latter equation, *P*(θ) denotes the probability of sampling a test taker with numerical IQ θ. Equation (9) assumes a discrete latent variable. In most applications, however, the hidden latent variable is modeled as continuous. In these cases the above summation has to be replaced by an integral with respect to *G*, the cumulative density function of θ. Nearly all applications specify a normal distribution for the latter.

Note that Equation (9) provides us with a frequency statement: In a sample of size *n* of test takers from ${\text{𝒫}}_{t}$, we expect *n*×*P*(*y*_*i*, 1_ = 1, *y*_*i*, 2_ = 0, …*y*_*i, k*_ = 0) test takers to show this particular response pattern according to our specified model. Stated differently, given estimates for the unknown parameters, e.g., item difficulties, item discrimination and variance of the latent variable, which enter Equations (9) through (8), we can plug in these estimates into the right hand side and evaluate the model fit via some discrepancy measure between the observed frequency count and the expected count according to the model.^3^

From the above outline, the following may be deduced. Firstly, as the computation of the marginals in (9) also involves *G*, an IRT model can show misfit despite a correct specification of the dimensionality of θ and of each IRF. This misfit is then solely caused by an incorrectly specified distribution function of the latent variable (i.e., of the random effect). Secondly, it is difficult to construct test statistics which allow for a detailed analysis of the cause of misspecification. That is, although we may observe a practically meaningful deviation of the observed and expected counts, we may not know if the latter is a result of the misspecification of the IRFs or of the distribution function. And thirdly, it follows from the first aspect that the model fit is highly dependent on subpopulations. That is, given two populations which only differ in the distribution of the latent ability, the appropriateness of the IRT model will be evaluated differently. In essence, this is already highlighted in Equation (9). That is, according to the law of total probability, (marginal) probabilities are always affected by the marginal distribution of the partitioning random variable (θ in this case) and differ from each other—even if all conditional distributions are identical.

We may further elaborate on the latter point: Assuming a validly constructed numerical IQ scale in accordance with the usual assumptions (entailing the normality of θ), it follows that we are likely to encounter nonnormality in subpopulations. For example, if we have a mixture of two subpopulations which differ in the location or variance of the latent ability (the analog reasoning as given in Section 4.1 applies). Likewise, if there is a variance restriction such as using the scale for job selection tasks, wherein the job applicants are supposed to show less variation in the IQ due to the requirements of the job profile (e.g., engineers; cf. Section 3). Both cases depict a simple, practical relevant mechanism which dissolves any prior existing normality. In conjunction with the second example, it follows that two researchers which examine the same scale in different (sub)populations are likely to disagree on the fit of the model solely due to a strong assumption on the distribution for θ.

Importantly, it must be emphasized that the outlined results also appear in other GLMM type models. Every GLMM model requires the specification of the distribution of an unobservable latent quantity.

## 5. Minimizing the risk of biased inferences

There is a fine line in reaching valid conclusions, with any violation of an assumption along the way potentially leading to biased conclusions. However, there are also strategies for dealing with the potential problems discussed in this paper. A scientifically sound approach to empirical research is, first, to be aware of the assumptions underlying the selection and analysis steps and, second, to explicitly state the assumptions and justify their validity. Both aspects require the following triad: A sufficiently developed theory, appropriate methods to generate and analyze the data, and a reliable body of relevant empirical studies. Appropriateness of the methods in turn implies availability and good knowledge of the adopted statistical techniques. All three components are necessarily interdependent and ideally evolve iteratively as knowledge is accumulated.

It follows from the foregoing sections that the maturity of a theory determines how precisely ${\text{𝒫}}_{△}$ and DGP Δ can be defined. The more developed a theory, the better informed a possible sampling design, the better justified the statistical analysis tools, and consequently the fewer untestable assumptions required. This in turn increases the credibility of inferences and helps to built better theories. Therefore, at any point in the process, available knowledge should be used to challenge, sharpen and develop a theory. In general, however, the systematic development of theories does not seem to have been given a high priority in psychological research (e.g.,Meehl, 1978; Fiedler, 2014; Eronen and Bringmann, 2021; McPhetres et al., 2021; Szollosi and Donkin, 2021). In the usual case, where the definition of the target population is vague at best, conclusions should be interpreted with great caution and perhaps limited to a smaller, defensible subpopulation, such as a group of students in a particular subject and age group.

A crucial condition for ignoring the selection mechanism resembles the fundamental condition in experimental settings to avoid systematic effects of confounding variables: Selection into the “observed” vs. “not observed” conditions may depend on observed covariates but not additionally on the outcome. In many psychological studies, this is implicitly assumed without further justification, but in order to allow compensation of a possible selectivity of an observed subsample and thus to justify statements about a broader subpopulation or even ${\text{𝒫}}_{t}$, the selection mechanism, the relevant variables and their relationships with the DGP Δ must be known. Thus, in addition to the theory of interest, at least a rudimentary auxiliary theory of response behavior must be available.

Based on not necessarily exact replications of a study, knowledge of response behavior can be built up iteratively by collecting variables informative of non-response. This can consist of individual information about non-respondents such as age or cohort membership in terms of age groups, field of study if units are students, or residential area (e.g., Groves et al., 2001). Although trying to collect this additional information requires more expensive data collection methods, it would allow researchers to adopt a weighting strategy, to include a correction term in the estimated model, to apply a (full information) maximum likelihood method or to generate multiple imputations to compensate for missing units (e.g.,Rubin, 1987; Robins et al., 1995; Schafer and Graham, 2002; Wooldridge, 2002, 2007, 2010). To allow valid inference, all these techniques require, in addition to more or less strong modeling assumptions, that all variables relevant to the non-response process are included in the analysis.

In addition to variables directly related to DGP Δ or response behavior, variables could be collected for explanatory purposes to help build an increasingly strong foundation by sharpening the definition of ${\text{𝒫}}_{t}$, helping to learn about possible mixture populations, and thus increasing knowledge about DGP Δ. The necessary exploratory analyses should be incentivized by publishing these as independent, citable articles. Similarly, research on the reasons for non-response should be encouraged to provide the research community with information on variables to compensate for unobserved units in related contexts.

If the theory underlying a research question of interest does not justify the assumptions necessary for the adopted analysis method, or if empirical results raise doubts whether they are met, then a sensitivity analysis, a multiverse analysis (Steegen et al., 2016) or the adaption of a robust or non-parametric estimation method may be an appropriate choice. The basic idea of sensitivity analyses is to analyze the data set at hand under a range of plausible assumptions. If inferences do not change substantially, they are robust with respect to this set of plausible assumptions (e.g., Rosenbaum and Rubin, 1983; in the context of missing values, see Rubin, 1987). This strategy, although not new, has not received much attention in applied research.

However, there is a way around using parametric models based on strong assumptions. Semi- or non-parametric methods require larger, although not necessarily much larger samples (e.g.,Spiess and Hamerle, 2000) but also less detailed formulated theories, which is helpful at an earlier stage of theory development. If then a random sample is selected from a clearly defined subpopulation according to a known sampling design and auxiliary variables are surveyed to compensate for possible selectivity due to non-response, the results may cautiously be interpreted with respect to the addressed subpopulation if model diagnostics following the analyses do not imply serious violation of assumptions. Of course, the whole procedure including all the variables surveyed should be described in detail and the data should be made publicly available to allow replications and evaluation of the results.

Semiparametric approaches, requiring less strong assumptions have been proposed, e.g., in biometrics and econometrics, respectively. Hansen (1982) proposes a generalized methods of moments (GMM) approach and Liang and Zeger (1986) a generalized estimating equations (GEE) approach. For valid inferences in (non-) linear (panel or repeated measurement) regression models, both approaches require only correct specification of the fixed part of a model, whereas the covariance structure may be misspecified. GMM is more flexible as it allows the estimation of more general models than GEE, but the latter is easier to use. Both approaches have been adapted or generalized since the 80's, e.g., to deal with many different situations, e.g., high dimensional data (Fan and Liao, 2014), panel or repeated measurement models with mixed continuous and ordinal outcomes (Spiess, 2006) or ordered stereotpye models (Spiess et al., 2020). Another approach that allows modeling linear or much more general, smooth non-linear effects of covariates on the mean and further shape parameters of the (conditional) outcome distribution is described in Rigby and Stasinopoulos (2005). This approach would be helpful when the effects of some covariates cannot be assumed to be linear, but need to be controlled.

For the non-parametric modeling approach, we limit ourselves to an example from IRT to illustrate that these arguably more robust approaches have been available, but have not been adopted by researchers in psychology: The theoretical underpinnings of some non-parametric approaches were established as early as the 1960s (Esary et al., 1967). One of the first practical outlines of a non-parametric approach to IRT was then given in the early 1970s by Mokken (1971), and some important generalizations of the latter—both in practical and theoretical aspects—were established in the 1980s (e.g., Holland and Rosenbaum, 1986) and 1990s (e.g., Ramsay, 1991). These results generally provide robustness against misspecification of the distribution of θ as well as misspecification of the IRFs. In many cases, *G* does not need to be specified at all, and the only relevant property of the IRF is monotonicity. Of course, this comes at a price, e.g., inference of the latent variable is done via simple sum scores. However, since the latter is already dominant in practical applications, this does not seem to be a severe restriction in practice.

Obviously, semi- or non-parametric approaches make less strong assumptions than fully parametric approaches, by allowing certain aspects of the statistical models to be miss- or unspecified. Besides the fact that they usually require more observations than fully parametric approaches, inferences about the misspecified aspects are either not possible or should be drawn very cautiously, e.g., when a correlation matrix might be misspecified. If no theory is available to justify a statistical model, including assumptions, a better strategy, if possible, would be to use a simpler design in conjunction with a simple and robust evaluation method (e.g.,Peterson, 2009). A notable side-effect of relying on simple designs and analysis steps is the availability of sufficiently elaborated tools for model diagnostics.

## 6. Discussion and conclusions

The methodological framework presented in Section 2 highlights the close linkage between scientific theory, sampling and data collection design as well as the statistical methods and models adopted to empirically test the theory. Since not much resources are devoted to the proper sampling of subjects from a well-defined population and since missing data are oftentimes ignored or assumed to follow a convenient missing mechanism, it can be assumed that assumptions of the commonly used parametric models are often violated. As shown in Section 3, the consequences can range from marginal biases to, e.g., in case of confidence intervals, actual coverage rates of true values close to zero even in the analysis of experimental data. It should also be noted that the outlined methodological problems cannot be prevented by preregistration or a ban on null hypothesis testing, nor can they be uncovered by mere replications within the same or very similar subpopulations. Increasing sample sizes, e.g., via online data collection, makes things even worse: the biases in the estimators do not vanish but the standard errors tend to zero, further lowering the actual coverage rates of confidence intervals in case of biased estimators.

Interestingly, although the approaches described in Section 5 circumvent severe problems in the estimation of general regression and IRT models, they seem to have largely been ignored. Instead, applied research seems to stick to convenience samples and highly specific (and fragile) parametric models. Among other reasons, such as publication policies, part of the problem may be that statistical training in psychology largely neglects sampling theory (e.g.,Särndal et al., 1992) (beyond sample size determination), strategies of avoiding or compensating for non-response (e.g., Rubin, 1987; Wooldridge, 2010) and problems of model misspecification.

However, the problem of missing reported model checks seems to be mainly caused by two factors. Firstly, in many modeling classes there is not a uniquely defined and accepted way of testing the modeling assumptions. In fact, the number of potential applicable statistics can be arbitrarily large. For example, assessing unidimensionality in an IRT model with *J* items can entail more than 10^*J*^ potential statistics (Ligtvoet, 2022) and there is no universal way to check unidimensionality. In conjunction with the dominance of parametric models this contributes to the fragility of the analysis.

Secondly, there is also an important connection of the lack of model checking with respect to the so called “garden of folking paths” (Gelman and Loken, 2014). The latter describes a sequence of data-dependent choices a researcher undertakes in order to arrive at his/her final analysis result. At each step, another decision could have been made with potential consequences for the outcome of the analysis. The mere fact that these decisions are not set a priori but are made data dependent contributes to the inflated effect sizes reported in the literature. Now suppose a researcher did arrive at a final result that seems to make sense in terms of content. In this case, we would argue that looking at additional model checks has already become highly unlikely. Not only is there the potential to “ruin” the result, but there is also the implication of going back to the drawing board and starting from scratch.

A potential way to resolve the problem of forking paths is given by preregistration of the study and by specifying the analysis protocol ahead of looking at the data. However, if we were humble with respect to the validity of our proposed model in the preregistration step, our plan would need to entail the possibility of misspecification. In some cases this could very well be incorporated in the preregistration step (e.g., Nosek et al., 2018). However, for complex types of analysis, the potential ways of model failures and the number of alternative models grows very fast, so that preregistration is unlikely to cover all potential paths of analysis. Furthermore, if it is suspected that the observed sample is selective and model diagnostics are considered as an important part of analysis, we must be open to sometimes unforeseen changes in the analysis plan—for otherwise we put too much trust in our models. This reveals that some proposals, such as preregistration, that aim to increase the trustworthiness of scientific research face additional major challenges, as the data dependence of the analysis may require switching to alternative models or procedures.

A longer-term strategy to overcome the shortcomings discussed above would be to hopefully increase students' appreciation of statistics by emphasizing the close interaction of theory, methods, and empirical information. A simple example would be to ask students to try to define the humans about which inferences are being made, to compare this definition with observed samples described in research papers, and to try to verbalize as clearly as possible the rationale and necessary assumptions for the inferences from the latter to the former. This exercise may also demonstrate that the validity of inferences depends on the weakest link in the chain. In addition, rather than teaching statistics as a clickable toolbox with many different models and techniques, and in addition to topics such as sample selection and missing data compensation, it may be beneficial to treat in depth the consequences of violated assumptions of standard techniques and models. The consequences of violated assumptions could be illustrated by simulating data sets following a real example, varying the assumptions being violated and discussing the consequences with respect to the inferences. To clearly demonstrate the consequences, this amounts to running simulation experiments. Students should learn that violation of some assumptions may have only mild consequences, whereas inferences can be very misleading if other assumptions are violated. Application of robust methods could be illustrated by applying semi- or non-parametric methods to a real problem for which the data set is available and compare the results with those reported in the corresponding research paper. Although the described problem-oriented strategy relies on practical examples and illustrations (or simulations), the corresponding theoretical concepts should be treated as well to a mathematical level such that the key ideas can be understood. Generalizations to more complex models should then be possible for students even without recourse on simple but often superficial receipts.

## Author contributions

MS: Conceptualization, Formal analysis, Visualization, Writing—original draft, Writing—review and editing. PJ: Conceptualization, Formal analysis, Writing—original draft, Writing—review and editing.

## Footnotes

^1^In fact, the CTT counterparts can be subsumed under the linear mixed model formulation, so that most of the following discussion applies also to the CTT framework.

^2^The local independence assumption is the formal manifestation of the statement that once the numerical IQ is fixed, the items show no statistical dependency anymore.

^3^There are some complications regarding the proper asymptotic behavior if the resulting table is sparse. Hence, our description is somewhat imprecise, as the correct setup would involve a properly defined likelihood function.
