# Supplementary material for: In models we trust: preregistration, large samples, and replication may not suffice
Source: Front Psychol. 2023 Sep 21;14:1266447. doi: 10.3389/fpsyg.2023.1266447 (PMC10551181; doi:10.3389/fpsyg.2023.1266447)
Supplement: Supplementary file 1 [file Data_Sheet_1.PDF]

frontiers

Supplementary Material

1 THE STANDARD ASSUMPTIONS IN LINEAR MODELS

The standard assumptions associated with the class of linear models are the Gauss-Markov assumptions, usually stated for $i, i' = 1, \dots$ and $i \neq i'$ as follows (e.g., Harville, 2018):

GM1) The dependency of the conditional means of the outcome has been correctly specified and follows the form:

$$\mathbb{E}(y_i|\boldsymbol{X}) = \mathbb{E}(y_i|\boldsymbol{x}_i) = \boldsymbol{x}_i^T\boldsymbol{\beta} \ .$$

GM2) The conditional variance of the outcome is independent of the predictors, i.e.

$$\text{var}(y_i|\boldsymbol{X}) = \sigma^2 \ .$$

GM3) The conditional covariance of each pair of outcomes is independent of the predictors, i.e.

$$\text{cov}(y_i, y_{i'}|\boldsymbol{X}) = 0 \ .$$

An additional assumption often made to justify calculating confidence intervals and conduction test in finite samples is the assumption:

NV) The conditional distribution (given $\boldsymbol{X}$) of y_i is normal.

Note that these assumptions are supposed to hold conditional on the x-values realized in the sample, but are often tacitly assumed to hold for all N elements in $\mathcal{P}_t$, an assumption we adopt in the main paper and this supplement.

2 SAMPLING AND RESPONSE MECHANISM

2.1 The General Framework: The Formal Side

Let $\boldsymbol{s}$ be a selection variable with elements s_i, $i = 1, \dots, N$, wherein s_i indicates whether the ith element of $\mathcal{P}_t$ is selected into the gross sample ($s_i = 1$) or not ($s_i = 0$) and $\boldsymbol{r}$ a response variable with elements r_i wherein $r_i = 1$ indicates whether the ith element is observed in the net sample and $r_i = 0$ if not. We assume throughout a monotone pattern of selection and response: If $r_i = 1$, then $s_i = 1$.

The sampling mechanism, in the survey literature denoted as sampling design, is represented by the probability function $g_{\boldsymbol{\gamma}_s}(\boldsymbol{s}|\boldsymbol{y}, \boldsymbol{x})$ and the response mechanism conditional on selection into the gross sample by the probability function $g_{\boldsymbol{\gamma}_r}(\boldsymbol{r}|\boldsymbol{s}, \boldsymbol{y}, \boldsymbol{x})$, wherein $\boldsymbol{\gamma}_s$ and $\boldsymbol{\gamma}_r$ are unknown parameters and $\boldsymbol{x}$ is a vectorized version of the matrix $\boldsymbol{X}$ of explanatory variables, where the constant term is omitted. Because a unit is only observed in the net sample if both $s_i = 1$ and $r_i = 1$, we integrate both mechanisms into one process for simplicity but without loss of generality, $g_{\boldsymbol{\gamma}}(\boldsymbol{v}|\boldsymbol{y}, \boldsymbol{x})$, wherein for each element of $\boldsymbol{v}$, $v_i = s_i r_i$, and $\boldsymbol{\gamma}$ is the unknown parameter governing this process. Hence, y_i and $\boldsymbol{x}_i$ are either both observed or they are not observed. Note that we do not consider item nonresponse.

Let $\boldsymbol{y}_{\text{mis}}$ denote the vector of variables y_i not observed and $\boldsymbol{y}_{\text{obs}}$ that part of $\boldsymbol{y}$ which is selected into the sample and observed. Correspondingly, define $\boldsymbol{x}_{\text{mis}}$ and $\boldsymbol{x}_{\text{obs}}$.

Starting point for inferences is usually the conditional distribution $f_{\boldsymbol{\theta}}(\boldsymbol{y}|\boldsymbol{x})$, assumed to hold in the population of interest, wherein $\boldsymbol{\theta}$ is an unknown parameter. This conditional distribution, or aspects thereof, i.e. usually the effect of $\boldsymbol{x}$ on $\boldsymbol{y}$, is assumed to describe the relevant relations of the DGP$_\Delta$. Note that $\boldsymbol{y}$ and $\boldsymbol{x}$ could be vectors of continuous or discrete variables or a mixture thereof. For simplicity, but without loss of generality, we assume that $\boldsymbol{y}$ is continuous if not otherwise noted.

A reasonable assumption often adopted is that, for all $i = 1, \ldots, N$, the scalar or vector-valued dependent variable $\boldsymbol{y}_i$ may depend on $\boldsymbol{x}_i$ but not in addition on $\boldsymbol{y}_{i'}$ or $\boldsymbol{x}_{i'}$, wherein $i \neq i'$. This implies $f_{\boldsymbol{\theta}}(\boldsymbol{y}|\boldsymbol{x}) = f_{\boldsymbol{\theta}}(\boldsymbol{y}_{\text{obs}}|\boldsymbol{x}_{\text{obs}}) f_{\boldsymbol{\theta}}(\boldsymbol{y}_{\text{mis}}|\boldsymbol{x}_{\text{mis}})$. We also assume that the model of the selection process is the same for all i.

Given a selected and observed sample of size n_{obs} which is generally much smaller than N, and adopting a classical model based frequentist statistical approach, the model often adopted is $f_{\boldsymbol{\theta}}(\boldsymbol{y}|\boldsymbol{x})$ using only those variables whose values have been observed, with $\boldsymbol{x}$ and n_{obs} fixed at their observed values. What actually should then be modeled, however, is the distribution of $\boldsymbol{y}_{\text{obs}}$ conditional on $\boldsymbol{x}_{\text{obs}}$ and $\boldsymbol{v}$, the pattern of observed and unobserved units, but marginal with respect to $\boldsymbol{y}_{\text{mis}}$,

$$\int \frac{g_\gamma(\boldsymbol{v}|\boldsymbol{y},\boldsymbol{x}) f_{\boldsymbol{\theta}}(\boldsymbol{y}|\boldsymbol{x})}{\int g_\gamma(\boldsymbol{v}|\boldsymbol{y},\boldsymbol{x}) f_{\boldsymbol{\theta}}(\boldsymbol{y}|\boldsymbol{x})\, d\boldsymbol{y}}\, d\boldsymbol{y}_{\text{mis}} \,, \tag{S1}$$

wherein the denominator is assumed to be not zero (cf. Rubin, 1976). This model takes the selection process explicitly into account and allows studying its effect on the model to be estimated.

In case of a classical model based frequentist statistical approach, inferences are evaluated on a hypothetically infinite number of repetitions of exactly the same random experiments (cf. Rubin, 1976). Assume

$$g_\gamma(\boldsymbol{v}_i|\boldsymbol{y}_i,\boldsymbol{x}_i,\boldsymbol{v}_{i'},\boldsymbol{y}_{i'},\boldsymbol{x}_{i'}) = g_\gamma(\boldsymbol{v}_i|\boldsymbol{y}_i,\boldsymbol{x}_i) \quad \text{for all } i \neq i' \,.$$

Then (S1) can be written as

$$\begin{aligned}
&\int \frac{g_\gamma(\boldsymbol{v}|\boldsymbol{y},\boldsymbol{x}) f_{\boldsymbol{\theta}}(\boldsymbol{y}|\boldsymbol{x})}{\int g_\gamma(\boldsymbol{v}|\boldsymbol{y},\boldsymbol{x}) f_{\boldsymbol{\theta}}(\boldsymbol{y}|\boldsymbol{x})\, d\boldsymbol{y}}\, d\boldsymbol{y}_{\text{mis}} \\
&\quad = \frac{g_\gamma(\boldsymbol{v}_{\text{obs}}|\boldsymbol{y}_{\text{obs}},\boldsymbol{x}_{\text{obs}}) f_{\boldsymbol{\theta}}(\boldsymbol{y}_{\text{obs}}|\boldsymbol{x}_{\text{obs}}) \ \int g_\gamma(\boldsymbol{v}_{\text{mis}}|\boldsymbol{y}_{\text{mis}},\boldsymbol{x}_{\text{mis}}) f_{\boldsymbol{\theta}}(\boldsymbol{y}_{\text{mis}}|\boldsymbol{x}_{\text{mis}})\, d\boldsymbol{y}_{\text{mis}}}{\int g_\gamma(\boldsymbol{v}|\boldsymbol{y},\boldsymbol{x}) f_{\boldsymbol{\theta}}(\boldsymbol{y}|\boldsymbol{x})\, d\boldsymbol{y}} \\
&\quad = \frac{g_\gamma(\boldsymbol{v}_{\text{obs}}|\boldsymbol{y}_{\text{obs}},\boldsymbol{x}_{\text{obs}}) f_{\boldsymbol{\theta}}(\boldsymbol{y}_{\text{obs}}|\boldsymbol{x}_{\text{obs}})}{\int g_\gamma(\boldsymbol{v}_{\text{obs}}|\boldsymbol{y}_{\text{obs}},\boldsymbol{x}_{\text{obs}}) f_{\boldsymbol{\theta}}(\boldsymbol{y}_{\text{obs}}|\boldsymbol{x}_{\text{obs}})\, d\boldsymbol{y}_{\text{obs}}} \,.
\end{aligned}$$

1. If $g_\gamma(\boldsymbol{v}_{\text{obs}}|\boldsymbol{y}_{\text{obs}},\boldsymbol{x}_{\text{obs}}) = g_\gamma(\boldsymbol{v}_{\text{obs}}|\boldsymbol{x}_{\text{obs}})$ or if $g_\gamma(\boldsymbol{v}_{\text{obs}}|\boldsymbol{y}_{\text{obs}},\boldsymbol{x}_{\text{obs}}) = g_\gamma(\boldsymbol{v}_{\text{obs}})$ for fixed $\boldsymbol{v}_{\text{obs}}$,

   $$\int \frac{g_\gamma(\boldsymbol{v}|\boldsymbol{y},\boldsymbol{x}) f_{\boldsymbol{\theta}}(\boldsymbol{y}|\boldsymbol{x})}{\int g_\gamma(\boldsymbol{v}|\boldsymbol{y},\boldsymbol{x}) f_{\boldsymbol{\theta}}(\boldsymbol{y}|\boldsymbol{x})\, d\boldsymbol{y}}\, d\boldsymbol{y}_{\text{mis}} = f_{\boldsymbol{\theta}}(\boldsymbol{y}_{\text{obs}}|\boldsymbol{x}_{\text{obs}}) \,,$$

   and the selection and unit response mechanism can be ignored.
2. If for fixed $\boldsymbol{v}$, $g_\gamma(\boldsymbol{v}|\boldsymbol{y},\boldsymbol{x})$ is the same for all observed values of $\boldsymbol{x}$ but may depend on variables affecting $\boldsymbol{y}$ which do not depend on $\boldsymbol{x}$, then it has been shown for specific models that inferences for regression

parameters based on $f_{\boldsymbol{\theta}}(\boldsymbol{y}_{\text{obs}}|\boldsymbol{x}_{\text{obs}})$ may be valid (e.g. Heckman, 1979; Terza, 1998; McCulloch, Neuhaus and Olin, 2016).

3. In all other cases, i.e. if the selection and unit response mechanism is a function of $\boldsymbol{x}$ and $\boldsymbol{y}$ then inferences based on $f_{\boldsymbol{\theta}}(\boldsymbol{y}_{\text{obs}}|\boldsymbol{x}_{\text{obs}})$ must be expected to be biased.

2.2 Examples and Figures

Figure 1 was generated with the TikZ and PGF Packages, version 3.1.5b (Tantau, 2020). Figures 2, 3a, 3b and 4 where generated with R, version 4.2.1 (R Core Team, 2022, RRID:SCR_001905).

2.2.1 One Measurement per Unit

For a clear presentation we ignore conditioning on the observed x- and z-variables in the following subsections. Note that $\boldsymbol{v}_{\text{obs}} := \mathbf{1}$, wherein $\mathbf{1}$ is a $(n_{\text{obs}} \times 1)$-vector of ones.

The least squares estimator ignoring the selection and response mechanism is given by

$$\hat{\boldsymbol{\beta}} = (\boldsymbol{X}^T\boldsymbol{X})^{-1}\boldsymbol{X}^T\boldsymbol{y}$$

with expected value

$$\begin{aligned}\mathbb{E}(\hat{\boldsymbol{\beta}}|\boldsymbol{v}_{\text{obs}}) &= \mathbb{E}((\boldsymbol{X}^T\boldsymbol{X})^{-1}\boldsymbol{X}^T\boldsymbol{y}|\boldsymbol{v}_{\text{obs}}) = \mathbb{E}((\boldsymbol{X}^T\boldsymbol{X})^{-1}\boldsymbol{X}^T(\boldsymbol{X}\boldsymbol{\beta} - \rho_{\epsilon,w}\sigma_\epsilon\boldsymbol{\lambda} + \tilde{\boldsymbol{\epsilon}})|\boldsymbol{v}_{\text{obs}}) \\ &= \boldsymbol{\beta} - \rho_{\epsilon,w}\sigma_\epsilon\,(\boldsymbol{X}^T\boldsymbol{X})^{-1}\boldsymbol{X}^T\boldsymbol{\lambda}\end{aligned}$$

and, if only one explanatory variable is considered, to

$$\mathbb{E}(\hat{\boldsymbol{\beta}}|\boldsymbol{x}_{\text{obs}}, \boldsymbol{v}_{\text{obs}}) = \boldsymbol{\beta} - \rho_{\epsilon,w}\sigma_\epsilon \begin{pmatrix} \bar{\lambda} - \bar{x}\tilde{s}_{\lambda,x}\tilde{s}_x^{-2} \\ \tilde{s}_{\lambda,x}\tilde{s}_x^{-2} \end{pmatrix} , \tag{S2}$$

wherein all involved parameters are evaluated at their true values, $\bar{\lambda}$ is the sample mean of $\lambda_1, \ldots, \lambda_{n_{\text{obs}}}$, $\tilde{s}_x^2$ is the sample variance of x_i and $\tilde{s}_{\lambda,x}$ is the sample covariance of λ_i and x_i values. Thus $\hat{\boldsymbol{\beta}}$ will be biased if both, the correlation of the errors w_i and ϵ_i of both processes, (1) and (2), and the correlation of λ_i and x_i in the observed part of the sample is not zero.

The variance of $\hat{\boldsymbol{\beta}}$ in a model with explanatory variables $\boldsymbol{X}$ is given by

$$\begin{aligned}\text{Var}(\hat{\boldsymbol{\beta}}|\boldsymbol{v}_{\text{obs}}) &= \sigma_\epsilon^2(\boldsymbol{X}^T\boldsymbol{X})^{-1} \\ &\quad - \rho_{\epsilon w}^2\sigma_\epsilon^2(\boldsymbol{X}^T\boldsymbol{X})^{-1}\boldsymbol{X}^T\text{diag}(\psi_1\lambda_1 + \lambda_1^2, \ldots, \psi_{n_{\text{obs}}}\lambda_{n_{\text{obs}}} + \lambda_{n_{\text{obs}}}^2)\boldsymbol{X}(\boldsymbol{X}^T\boldsymbol{X})^{-1},\end{aligned}$$

wherein diag$(\cdot)$ denotes a diagonal matrix, and can be estimated by

$$\widehat{\text{Var}}(\hat{\boldsymbol{\beta}}|\boldsymbol{v}_{\text{obs}}) = (\boldsymbol{X}^T\boldsymbol{X})^{-1}\boldsymbol{X}^T\text{diag}(e_1^2, \ldots, e_{n_{\text{obs}}}^2)\boldsymbol{X}(\boldsymbol{X}^T\boldsymbol{X})^{-1} ,$$

wherein $e_i = (y_i - \boldsymbol{x}_i^T\hat{\boldsymbol{\beta}})$ is the residual of the ith unit. This estimator is different from the standard variance estimator which also ignores the selection process,

$$\hat{\sigma}_{\text{error}}^2\,(\boldsymbol{X}^T\boldsymbol{X})^{-1}, \text{ wherein } \hat{\sigma}_{\text{error}}^2 = \frac{1}{n_{\text{obs}} - p}\Big(\sum_{i=1}^{n_{\text{obs}}} e_i^2\Big)$$

and in addition assumes equal error variances across all observed units. Thus, this variance estimator will lead to standard errors that can not be expected to be correct.

For one binary explanatory variable x_i, the estimator ignoring the selection process and its variance simplify to

$$\hat{\boldsymbol{\beta}} = \begin{pmatrix} \bar{y}_0 \\ \bar{y}_1 - \bar{y}_0 \end{pmatrix} , \quad \text{with expected value given by (6) of the main text ,} \tag{S3}$$

$$\begin{aligned} \mathrm{Var}(\hat{\boldsymbol{\beta}}|\boldsymbol{v}_{\text{obs}}) = & \frac{\sigma_\epsilon^2}{n_0} \begin{pmatrix} 1 & -1 \\ -1 & n/n_1 \end{pmatrix} \\ & - \rho_{\epsilon w}^2 \sigma_\epsilon^2 \begin{pmatrix} n_0^{-1}(\overline{\psi\lambda + \lambda^2})_0 & -n_0^{-1}(\overline{\psi\lambda + \lambda^2})_0 \\ -n_0^{-1}(\overline{\psi\lambda + \lambda^2})_0 & n_0^{-1}(\overline{\psi\lambda + \lambda^2})_0 + n_1^{-1}(\overline{\psi\lambda + \lambda^2})_1 \end{pmatrix} , \end{aligned} \tag{S4}$$

wherein $\bar{y}_0$ and $\bar{y}_1$ are the means of y-values, $(\overline{\psi\lambda + \lambda^2})_0$ and $(\overline{\psi\lambda + \lambda^2})_1$ are the means of $\psi\lambda + \lambda^2$-values for which $x = 0$ and $x = 1$, n_0 and n_1 are the number of units with $x = 0$ and $x = 1$, respectively.

Allowing the error-variances to differ across the two different conditions, requires modeling the effects of the selection process separately for the two conditions, i.e.

$$y_{j,i} = \boldsymbol{x}_{j,i}^T \boldsymbol{\beta} - \rho_{\epsilon,w} \sigma_{\epsilon_j} \lambda_i + \tilde{\epsilon}_{j,i} ,$$

wherein $j = 0, 1$ denotes the condition $x = 0$ and $x = 1$, and $\mathbb{E}(\tilde{\epsilon}_{j,i}|v_i = 1) = 0$ for $j = 0, 1$, respectively (cf. (4) of the main text). Then the expected value of the estimator ignoring selection into the observed part of the sample is

$$\mathbb{E}(\hat{\boldsymbol{\beta}}|\boldsymbol{v}_{\text{obs}}) = \begin{pmatrix} \mu_0 \\ \mu_1 - \mu_0 \end{pmatrix} - \rho_{\epsilon,w} \begin{pmatrix} \sigma_{\epsilon_0} \bar{\lambda}_0 \\ \sigma_{\epsilon_1} \bar{\lambda}_1 - \sigma_{\epsilon_0} \bar{\lambda}_0 \end{pmatrix} , \quad \text{with variance} \tag{S5}$$

$$\begin{aligned} \mathrm{Var}(\hat{\boldsymbol{\beta}}|\boldsymbol{v}_{\text{obs}}) = & \frac{\sigma_{\epsilon_0}^2}{n_0} [1 - \rho_{\epsilon w}^2 (\overline{\psi\lambda + \lambda^2})_0] \begin{pmatrix} 1 & -1 \\ -1 & 1 \end{pmatrix} \\ & + \frac{\sigma_{\epsilon_1}^2}{n_1} [1 - \rho_{\epsilon w}^2 (\overline{\psi\lambda + \lambda^2})_1] \begin{pmatrix} 0 & 0 \\ 0 & 1 \end{pmatrix} . \end{aligned} \tag{S6}$$

2.2.2 One Measurement per Unit: Figure 3

The graphics in Figure 3 were generated as follows:

1. $n = 500$ values were generated for binary x_i, $i = 1, \ldots, n$, using a pseudorandom number generator for variables following a binomial distribution, with $\pi_x := \Pr(x_i) = 0.5$.
2. For graphic a), values for binary z_i were generated from a binomial distribution with $\Pr(z_i = 1|x_1) = 0.6 + 0.4x_i$ resulting in a correlation $r_{zx} = 0.5$ of z_i and x_i and marginal probability $\pi_z = \Pr(z_1 = 1) = 0.8$. For graphic b), z_i is not relevant because the corresponding entry in $\boldsymbol{\gamma}$ is set to zero.
3. Values for w_i were generated from a pseudorandom number generator that simulates values from a normal distribution with mean zero and variance $\sigma_w^2 = 1$.

4. Values for v_i were generated following (1) in the main text. For graphic a), $\boldsymbol{\gamma} = (0\ \gamma_2)^T$, wherein $\gamma_2 \in \{0.1, 0.25, 0.5, 0.75\}$, and for graphic b) $\boldsymbol{\gamma} = (0\ 0)^T$.
5. With $c = 0.3$, $\boldsymbol{z}_i = (1\ z_i)^T$, the above values for $\boldsymbol{\gamma}$ and $\sigma_w^2 = 1$, ψ_i, $\phi(\psi_i)$, $\Phi(\psi_i)$ and λ_i were calculated as described in Section 3.2 of the main text. The values for $\boldsymbol{\gamma}$ given above lead to values for δ_λ being equal to 0.026, 0.076, 0.177 and 0.312, respectively, in graphic a) and to $\delta_\lambda = 0$ in graphic b). The proportion of observed out of the $n = 500$ units varied between 0.212 and 0.354 for graphic a) and was 0.394 for graphic b). We could have simulated a much larger number of units n and a very small sampling fraction. However, given the simple setup with only one binary z_i-variable, the illustrative character of the graphics would have not changed substantially. For graphic a) the number observed units with $x_i = 0$ varied between $n_0 = 60$ and $n_0 = 88$ and with $x_i = 1$ varied between $n_1 = 46$ and $n_i = 89$, for graphic b) the corresponding numbers are $n_0 = 101$ and $n_1 = 96$.
6. For graphic a) we chose $\rho_{\epsilon,w} \in \{-0.99, -0.98, \ldots, 0.98, 0.99\}$ and $\sigma_\epsilon^2 = 1$, for graphic b) $\rho_{\epsilon,w} = 0.4$, $\sigma_{\epsilon_0}^2 \in \{0.25, 1, 4, 9\}$ and $\sigma_{\epsilon_1}^2 \in \{0.1, 0.15, \ldots, 9.95, 1\}$, respectively.
7. The rows of design matrix $\boldsymbol{X}$ are $\boldsymbol{x}_i^T = (1\ x_i)$ with x_i the binary variable from the first step. The true value of $\boldsymbol{\beta} = (\beta_0\ \beta_1)^T$ was set to $\boldsymbol{\beta} = (2\ 0)^T$, so that $\mathbb{E}(y_i|x_i = 1) - \mathbb{E}(y_i|x_i = 0) = \mu_1 - \mu_0 = 0$.
8. With the above specifications the expected values of $\hat{\boldsymbol{\beta}}$ can be calculated as given in (4) and (5) in the main text, and and the variances can be calculated as given in (S4) and (S6) in this supplement, respectively. The coverage is calculated as follows:

   To avoid unnecessary complexity, we assume the variance of $\hat{\boldsymbol{\beta}}$ to be known. Let $q_{\alpha/2}$ and $q_{1-\alpha/2}$ be the $\alpha/2$- and $1-\alpha/2$-quantiles of an appropriate distribution, $\text{sd}_{\hat{\beta}_1}$ the positive square root of the lower right entry in matrix $\text{Var}(\hat{\boldsymbol{\beta}}|\boldsymbol{v})$ (see (S4) and (S6)), 'bias' the bias of the estimator (see (4) and (5) of the main text) and $\beta_1 = \mu_1 - \mu_0$. Then

$$
\begin{aligned}
1-\alpha &= \Pr(q_{\alpha/2} \leq \frac{\hat{\beta}_1 - \mathbb{E}(\hat{\beta}_1|\boldsymbol{v})}{\text{sd}_{\hat{\beta}_1}} \leq q_{1-\alpha/2}) \\
&= \Pr(\frac{\hat{\beta}_1 - \mathbb{E}(\hat{\beta}_1|\boldsymbol{v})}{\text{sd}_{\hat{\beta}_1}} \leq q_{1-\alpha/2}) - \Pr(\frac{\hat{\beta}_1 - \mathbb{E}(\hat{\beta}_1|\boldsymbol{v})}{\text{sd}_{\hat{\beta}_1}} \leq q_{\alpha/2}) \\
&= \Pr(\frac{\hat{\beta}_1 - \beta_1}{\text{sd}_{\hat{\beta}_1}} - \frac{\text{bias}}{\text{sd}_{\hat{\beta}_1}} \leq q_{1-\alpha/2}) - \Pr(\frac{\hat{\beta}_1 - \beta_1}{\text{sd}_{\hat{\beta}_1}} - \frac{\text{bias}}{\text{sd}_{\hat{\beta}_1}} \leq q_{\alpha/2}) \;.
\end{aligned}
$$

   Given the large sample size and $\alpha = 0.05$, the probability that the confidence intervals with lower and upper limits ci_{lower} and ci_{upper}, ignoring selection,

$$
\text{ci}_{\text{lower}} = \hat{\beta}_1 - 1.96\,\text{sd}_{\hat{\beta}_1} \quad \text{and} \quad \text{ci}_{\text{upper}} = \hat{\beta}_1 + 1.96\,\text{sd}_{\hat{\beta}_1}\;,
$$

   cover the true value $\beta_1 = 0$ can be approximated by

$$
\Phi(1.96 + \frac{\text{bias}}{\text{sd}_{\hat{\beta}_1}}) - \Phi(-1.96 + \frac{\text{bias}}{\text{sd}_{\hat{\beta}_1}})
$$

   wherein $\Phi(a + \text{bias}/\text{sd}_{\hat{\beta}_1})$ is equal to the value of the distribution function of a normal distribution with mean $-\text{bias}/\text{sd}_{\hat{\beta}_1}$ and unit variance at the value a.

2.2.3 Two Measurements per Unit

Assume that for each unit i in an observed sample two dependent variables y are observed, so that $\boldsymbol{y}_i = (y_{i,0}\ y_{i,1})^T$ for all $i = 1, \ldots, n_{\text{obs}}$. Correspondingly, for each unit a $(2 \times p)$-covariate or design matrix $\boldsymbol{X}_i = (\boldsymbol{x}_{i,0}\ \boldsymbol{x}_{i,1})^T$ is observed, wherein $\boldsymbol{x}_{it}$ is the $(p \times 1)$ vector of covariates of the ith unit associated with the tth condition ($t = 0, 1$), including a degenerate variable with value one for the constant term.

Suppose that the true DGP of scientific interest in a population $\mathcal{P}_t$ at time point t is correctly modeled via selection mechanism (1) of the main text,

$$\boldsymbol{y}_i = \boldsymbol{X}_i\boldsymbol{\beta} + \boldsymbol{\epsilon}_i, \quad \text{wherein} \quad \boldsymbol{\epsilon}_i = (\epsilon_{i,0}\ \ \epsilon_{i,1})^T \text{ and}$$

$$\begin{pmatrix} \epsilon_{i,0} \\ \epsilon_{i,1} \\ w_i \end{pmatrix} \sim N\left[\begin{pmatrix} 0 \\ 0 \\ 0 \end{pmatrix}, \begin{pmatrix} \sigma^2_{\epsilon_0} & \sigma_{\epsilon_0,\epsilon_1} & \sigma_{\epsilon_0,w} \\ \sigma_{\epsilon_0,\epsilon_1} & \sigma^2_{\epsilon_1} & \sigma_{\epsilon_1,w} \\ \sigma_{\epsilon_0,w} & \sigma_{\epsilon_1,w} & \sigma^2_w \end{pmatrix}\right], \text{ independent from } \boldsymbol{X}_{i'}, \boldsymbol{z}_{i'}\ , i, i' = 1, \ldots, N\ .$$

Define ψ_i and λ_i as in Sections 3.2 and 3.2.1 of the main text, then, because $w_i | w_i \le c - \boldsymbol{z}_i^T\boldsymbol{\gamma}$ follows a right-truncated normal distribution,

$$\begin{aligned}
\mathbb{E}(\boldsymbol{y}_i | v_i = 1) &= \mathbb{E}(\boldsymbol{y}_i | v_i^* \le c) = \boldsymbol{X}_i\boldsymbol{\beta} - \sigma_w^{-1}\,\lambda_i \begin{pmatrix} \sigma_{\epsilon_0,w} \\ \sigma_{\epsilon_1,w} \end{pmatrix}\ , \\
\text{Var}(\boldsymbol{y}_i | v_i = 1) &= \text{Var}(\boldsymbol{y}_i | v_i^* \le c) \\
&= \begin{pmatrix} \sigma^2_{\epsilon_0} & \sigma_{\epsilon_0,\epsilon_1} \\ \sigma_{\epsilon_0,\epsilon_1} & \sigma^2_{\epsilon_1} \end{pmatrix} - \sigma_w^{-2}(\psi_i\lambda_i + \lambda_i^2)\begin{pmatrix} \sigma_{\epsilon_0,w} \\ \sigma_{\epsilon_1,w} \end{pmatrix}\begin{pmatrix} \sigma_{\epsilon_0,w} & \sigma_{\epsilon_1,w} \end{pmatrix}\ .
\end{aligned}$$

Thus, the model to be estimated in the subpopulation that can be observed is

$$\boldsymbol{y}_i = \boldsymbol{X}_i\boldsymbol{\beta} - \sigma_w^{-1}\,\lambda_i \begin{pmatrix} \sigma_{\epsilon_0,w} \\ \sigma_{\epsilon_1,w} \end{pmatrix} + \tilde{\boldsymbol{\epsilon}}_i\ , \tag{S7}$$

wherein $\mathbb{E}(\tilde{\boldsymbol{\epsilon}}_i | v_i = 1) = \boldsymbol{0}$ for all i, i' and the term $\sigma_w^{-1}\lambda_i\left(\begin{smallmatrix} \sigma_{\epsilon_0,w} \\ \sigma_{\epsilon_1,w} \end{smallmatrix}\right)$ corrects for a possible bias due to the selection and unit response mechanism. Note that λ_i is a function of $\boldsymbol{z}_i$ and so is the covariance matrix $\text{Var}(\boldsymbol{y}_i | v_i = 1)$. Further,

$$\sigma_w^{-1}\,\lambda_i \begin{pmatrix} \sigma_{\epsilon_0,w} \\ \sigma_{\epsilon_1,w} \end{pmatrix} = \lambda_i \begin{pmatrix} \rho_{\epsilon_0,w}\sigma_{\epsilon_0} \\ \rho_{\epsilon_1,w}\sigma_{\epsilon_1} \end{pmatrix}\ ,$$

wherein $\rho_{\epsilon_0,w}$ is the true correlation of $\epsilon_{i,0}$ and w, and $\rho_{\epsilon_1,w}$ is the true correlation of $\epsilon_{i,1}$ and w for all i, respectively.

Write

$$\boldsymbol{X} = \begin{pmatrix} \boldsymbol{X}_1 \\ \vdots \\ \boldsymbol{X}_{n_{\text{obs}}} \end{pmatrix} \quad \text{and} \quad \boldsymbol{y} = \begin{pmatrix} \boldsymbol{y}_1 \\ \vdots \\ \boldsymbol{y}_{n_{\text{obs}}} \end{pmatrix}\ .$$

Then, ignoring (self-)selection into the sample, the unknown parameter $\boldsymbol{\beta}$ could iteratively be estimated using the generalized least squares estimator

$$\hat{\boldsymbol{\beta}} = (\boldsymbol{X}^T\widehat{\boldsymbol{\Sigma}}^{-1}\boldsymbol{X})^{-1}\boldsymbol{X}^T\widehat{\boldsymbol{\Sigma}}^{-1}\boldsymbol{y} \ , \tag{S8}$$

wherein $\widehat{\boldsymbol{\Sigma}} = \text{bdiag}(\widehat{\boldsymbol{\Sigma}}_1, \ldots, \widehat{\boldsymbol{\Sigma}}_{n_{\text{obs}}})$, bdiag$(\cdot)$ denotes a block-diagonal matrix, and $\widehat{\boldsymbol{\Sigma}}_i$ is a covariance matrix estimated in each iteration using residuals $\boldsymbol{e}_i = (\boldsymbol{y}_i - \boldsymbol{X}_i\hat{\boldsymbol{\beta}})$ from the preceding step. Further, it can be shown that

$$\begin{aligned}\mathbb{E}(\hat{\boldsymbol{\beta}}|\boldsymbol{v}_{\text{obs}}) &= (\boldsymbol{X}^T\widehat{\boldsymbol{\Sigma}}^{-1}\boldsymbol{X})^{-1}\boldsymbol{X}^T\widehat{\boldsymbol{\Sigma}}^{-1}\mathbb{E}(\boldsymbol{y}|\boldsymbol{v}_{\text{obs}}) \\ &= \boldsymbol{\beta} - (\boldsymbol{X}^T\widehat{\boldsymbol{\Sigma}}^{-1}\boldsymbol{X})^{-1}\sum_{i=1}^{n_{\text{obs}}}\lambda_i \ \boldsymbol{X}_i^T\widehat{\boldsymbol{\Sigma}}_i^{-1}\begin{pmatrix}\rho_{\epsilon_0,w}\sigma_{\epsilon_0}\\ \rho_{\epsilon_1,w}\sigma_{\epsilon_1}\end{pmatrix} \ .\end{aligned} \tag{S9}$$

The variance of $\hat{\boldsymbol{\beta}}$ is

$$\begin{aligned}\text{Var}(\hat{\boldsymbol{\beta}}|\boldsymbol{v}_{\text{obs}}) = {} & (\boldsymbol{X}^T\widehat{\boldsymbol{\Sigma}}^{-1}\boldsymbol{X})^{-1}\boldsymbol{X}^T\widehat{\boldsymbol{\Sigma}}^{-1} \\ & \times \text{bdiag}(\text{Var}(\boldsymbol{y}_1|v_1 = 1), \ldots, \text{Var}(\boldsymbol{y}_{n_{\text{obs}}}|v_{n_{\text{obs}}} = 1)) \\ & \times \widehat{\boldsymbol{\Sigma}}^{-1}\boldsymbol{X}^T(\boldsymbol{X}^T\widehat{\boldsymbol{\Sigma}}^{-1}\boldsymbol{X})^{-1} \ ,\end{aligned}$$

wherein $\text{Var}(\boldsymbol{y}_i|v_i = 1)$ can be estimated by the robust or sandwich estimator

$$\widehat{\text{Var}}(\hat{\boldsymbol{\beta}}|\boldsymbol{v}_{\text{obs}}) = (\boldsymbol{X}^T\widehat{\boldsymbol{\Sigma}}^{-1}\boldsymbol{X})^{-1}\boldsymbol{X}^T\widehat{\boldsymbol{\Sigma}}^{-1}\text{bdiag}(\boldsymbol{e}_1\boldsymbol{e}_1^T, \ldots, \boldsymbol{e}_{n_{\text{obs}}}\boldsymbol{e}_{n_{\text{obs}}}^T)\widehat{\boldsymbol{\Sigma}}^{-1}\boldsymbol{X}^T(\boldsymbol{X}^T\widehat{\boldsymbol{\Sigma}}^{-1}\boldsymbol{X})^{-1} \ , \tag{S10}$$

which allows for heteroscedasticity of the error terms.

Hence, the estimator $\hat{\boldsymbol{\beta}}$ will generally be biased if the last term in (S9) does not vanish. In addition, the error variables in the observed sample are no longer normally distributed and the standard assumption of equal covariance matrices for all i will be violated in most cases.

The example considered in Section 3 of the main text is characterized by $\hat{\boldsymbol{\Sigma}}_i = \hat{\boldsymbol{\Sigma}}_{i'}$ for all i, i',

$$\boldsymbol{X}_i = \begin{pmatrix}1 & 0\\ 1 & 1\end{pmatrix} \quad \text{and} \quad \boldsymbol{X}_i^{-1} = \begin{pmatrix}1 & 0\\ -1 & 1\end{pmatrix} .$$

Then it can be shown that (S8) and (S10) simplify to

$$\hat{\boldsymbol{\beta}} = \begin{pmatrix}\bar{y}_0\\ \bar{y}_1 - \bar{y}_0\end{pmatrix} \quad \text{and} \quad \widehat{\text{Var}}(\hat{\boldsymbol{\beta}}|\boldsymbol{v}_{\text{obs}}) = \frac{1}{n_{\text{obs}}}\begin{pmatrix}\tilde{s}_0^2 & \tilde{s}_{0,1} - \tilde{s}_0^2\\ \tilde{s}_{0,1} - \tilde{s}_0^2 & \tilde{s}_0^2 - 2\tilde{s}_{0,1} + \tilde{s}_1^2\end{pmatrix} ,$$

wherein the means $\bar{y}_0$ and $\bar{y}_1$ are taken over observed values,

$$\begin{aligned}\tilde{s}_0^2 &= n_{\text{obs}}^{-1}\sum_{i=1}^{n_{\text{obs}}}(y_{i,0} - \boldsymbol{x}_{i,0}^T\hat{\boldsymbol{\beta}})^2 \ , \quad \tilde{s}_{0,1} = n_{\text{obs}}^{-1}\sum_{i=1}^{n_{\text{obs}}}(y_{i,0} - \boldsymbol{x}_{i,0}^T\hat{\boldsymbol{\beta}})(y_{i,1} - \boldsymbol{x}_{i,1}^T\hat{\boldsymbol{\beta}}) \quad \text{and} \\ \tilde{s}_1^2 &= n_{\text{obs}}^{-1}\sum_{i=1}^{n_{\text{obs}}}(y_{i,1} - \boldsymbol{x}_{i,1}^T\hat{\boldsymbol{\beta}})^2 \ ,\end{aligned}$$

respectively. Further, evaluating all parameters at their true values,

$$\mathbb{E}(\hat{\boldsymbol{\beta}}|\boldsymbol{v}_{\text{obs}}) = \boldsymbol{\beta} - \bar{\lambda}\begin{pmatrix} \rho_{\epsilon_0,w}\sigma_{\epsilon_0} \\ \rho_{\epsilon_1,w}\sigma_{\epsilon_1} - \rho_{\epsilon_0,w}\sigma_{\epsilon_0} \end{pmatrix}, \quad \bar{\lambda} = \frac{1}{n_{\text{obs}}}\sum_{i=1}^{n_{\text{obs}}} \lambda_i \quad \text{and} \tag{S11}$$

$$\begin{aligned} \text{Var}(\hat{\boldsymbol{\beta}}|\boldsymbol{v}_{\text{obs}}) &= \frac{1}{n_{\text{obs}}}\begin{pmatrix} \sigma^2_{\epsilon_0} & \sigma_{\epsilon_0,\epsilon_1} - \sigma^2_{\epsilon_0} \\ \sigma_{\epsilon_0,\epsilon_1} - \sigma^2_{\epsilon_0} & \sigma^2_{\epsilon_1} - 2\sigma_{\epsilon_0,\epsilon_1} + \sigma^2_{\epsilon_0} \end{pmatrix} \\ &\quad - \frac{\overline{\psi\lambda + \lambda^2}}{n_{\text{obs}}\sigma^2_w}\begin{pmatrix} \sigma^2_{\epsilon_0 w} & \sigma_{\epsilon_0,w}(\sigma_{\epsilon_1,w} - \sigma_{\epsilon_0,w}) \\ \sigma_{\epsilon_0,w}(\sigma_{\epsilon_1,w} - \sigma_{\epsilon_0,w}) & (\sigma_{\epsilon_1,w} - \sigma_{\epsilon_0,w})^2 \end{pmatrix}, \end{aligned} \tag{S12}$$

wherein $\overline{\psi\lambda + \lambda^2}$ is the mean over the sum of $\psi_i\lambda_i$ and λ_i^2 values of the observed units.

2.2.4 Two Measurements per Unit: Figure 4

Basically, Figure 4 was generated like the graphics in Figure 3 under R version 4.2.1. Thus, we mainly describe the differences:

1. For each of $n = 500$ units a matrix $\boldsymbol{X} = \left(\begin{smallmatrix} 1 & 0 \\ 1 & 1 \end{smallmatrix}\right)$ was generated.
2. The z_i variable had no effect on v_i^* (see below).
3. Values for w_i were generated as for Figure 3.
4. Values for v_i were generated following (1) of the main text with $\boldsymbol{\gamma} = (0\ 0)^T$.
5. With $c = -0.2$, $\boldsymbol{z}_i = (1\ z_i)^T$, the above values for $\boldsymbol{\gamma}$ and $\sigma_w^2 = 1$, ψ_i, $\phi(\psi_i)$, $\Phi(\psi_i)$ and λ_i were calculated as described in Section 3.2 of the main text. The value for $\boldsymbol{\gamma}$ given above leads to a mean value of $\bar{\lambda} = 0.9294$ for the λ_i values in the observed sample. The proportion of observed out of the $n = 500$ units was 0.41, so that $n_0 = n_1 = 205$.
6. We chose $\rho_{\epsilon_0,w} = 0$, $\rho_{\epsilon_1,w} \in \{-0.9, -.89, \ldots, 0.89, 0.9\}$, $\sigma^2_{\epsilon_0} = 1$, $\sigma^2_{\epsilon_1} \in \{0.25, 1, 4, 9\}$ and covariance of ϵ_0 and ϵ_1 as $\sigma_{\epsilon_0,\epsilon_1} = 0.2$.
7. Individual matrices $\boldsymbol{X}_i$ were stacked over each other to generate the design matrix $\boldsymbol{X}$. As for Figure 3, the true value of $\boldsymbol{\beta} = (\beta_0\ \beta_1)^T$ was set to $\boldsymbol{\beta} = (2\ 0)^T$.
8. With the above specifications the expected values and the variances of the estimators $\hat{\boldsymbol{\beta}}$ can be calculated as given in (S11) and (S12), respectively. The coverage is calculated as for Figure 3.

REFERENCES

Harville, D.A. (2018). *Linear Models and the Relevant Distributions and Matrix Algebra*. Taylor & Francis.

Heckman J. J. (1979). Sample Selection Bias as a Specification Error. *Econometrica, 47(1),* 153–161. doi:10.2307/1912352

McCulloch, C. E., Neuhaus, J. M., & Olin, R. L. (2016). Biased and Unbiased Estimation in Longitudinal Studies with Informative Visit Processes, *Biometrics 72,* 1315–1324. doi:10.1111/biom.12501

R Core Team (2022). *R: A Language and Environment for Statistical Computing*. R Foundation for Statistical Computing: Vienna, Austria. `https://www.R-project.org/`

Rubin, D. B. (1976). Inference and Missing Data. *Biometrika, 63(3),* 581–590. doi:10.1093/biomet/63.3.581

Tantau, T. (2020). *The TikZ and PGF Packages. Manual for version 3.1.5b.* `http://sourceforge.net/projects/pgf/`

Terza, J. V. (1998). Estimating count data models with endogenous switching: sample selection and endogenous treatment effects. *J. Econom. 84,* 129–154. doi:10.1016/S0304-4076(97)00082-1
